# Ligand-Based Drug Design of Genipin Derivatives with Cytotoxic Activity against HeLa Cell Line: A Structural and Theoretical Study

**DOI:** 10.3390/ph16121647

**Published:** 2023-11-23

**Authors:** Diana López-López, Rodrigo Said Razo-Hernández, César Millán-Pacheco, Mario Alberto Leyva-Peralta, Omar Aristeo Peña-Morán, Jessica Nayelli Sánchez-Carranza, Verónica Rodríguez-López

**Affiliations:** 1 Facultad de Farmacia, Universidad Autónoma del Estado de Morelos, Cuernavaca 62209, Mexico; diana.lopezl@uaem.edu.mx (D.L.-L.); cmp@uaem.mx (C.M.-P.); jessica.sanchez@uaem.mx (J.N.S.-C.); 2 Laboratorio de Quimioinformática y Diseño de Fármacos, Centro de Investigación en Dinámica Celular, Instituto de investigación en Ciencias Básicas y Aplicadas, Universidad Autónoma del Estado de Morelos, Cuernavaca 62209, Mexico; 3 Departamento de Ciencias Químico Biológicas y Agropecuarias, Universidad de Sonora, H. Caborca, Sonora 83621, Mexico; mario.leyva@unison.mx; 4 Departamento de Ciencias Farmacéuticas, División de Ciencias de la Salud, Universidad Autónoma del Estado de Quintana Roo, Chetumal 77019, Mexico; omar.moran@uqroo.edu.mx

**Keywords:** cytotoxic iridoids, SAR, DFT, dipole moment, QSAR, ligand-based design

## Abstract

Cervical cancer is a malignant neoplastic disease, mainly associated to HPV infection, with high mortality rates. Among natural products, iridoids have shown different biological activities, including cytotoxic and antitumor effects, in different cancer cell types. Geniposide and its aglycone Genipin have been assessed against different types of cancer. In this work, both iridoids were evaluated against HeLa and three different cervical cancer cell lines. Furthermore, we performed a SAR analysis incorporating 13 iridoids with a high structural similarity to Geniposide and Genipin, also tested in the HeLa cell line and at the same treatment time. Derived from this analysis, we found that the dipole moment (magnitude and direction) is key for their cytotoxic activity in the HeLa cell line. Then, we proceeded to the ligand-based design of new Genipin derivatives through a QSAR model (R^2^ = 87.95 and Q^2^ = 62.33) that incorporates different quantum mechanic molecular descriptor types (ρ, ΔPSA, ${∆Polarizability}^{2}$, and logS). Derived from the ligand-based design, we observed that the presence of an aldehyde or a hydroxymethyl in C4, hydroxyls in C1, C6, and C8, and the lack of the double bond in C7–C8 increased the predicted biological activity of the iridoids. Finally, ten simple iridoids (D9, D107, D35, D36, D55, D56, D58, D60, D61, and D62) are proposed as potential cytotoxic agents against the HeLa cell line based on their predicted IC_50_ value and electrostatic features.

## 1. Introduction

Cancer is a group of diseases characterized by abnormal cells that grow uncontrollably, and do not recognize cell death signaling [1]. Then, these abnormal cells can invade adjoining tissues and spread to other body organs in a process named metastasis. In 2020, 19 million new cases were diagnosed and 9.9 million deaths by cancer worldwide were reported for men and women, being the second cause of death [2].

Cervical cancer is a malignant neoplastic disease, originating in women’s cervixes. It is the fourth cause of incidence and death in women between 0 and 85 years globally, with 604 127 new cases and 341 831 deaths reported in 2020 [2]. Human papillomavirus (HPV) infection is the principal cause of cervical cancer. HPV16 and HPV18 are the two main high-risk HPVs related to cervical cancer development, and they are present in 70–75% of cervical cancer patient biopsies, with HPV16 the most carcinogenic subtype [3].

Since the actual chemotherapeutic treatments are not selective and, in many cases, cancer cells have developed resistance to them, finding new anti-cancer compounds with desirable selectivity toward cancer cells is crucial nowadays [4].

Vincristine, irinotecan, etoposide, and paclitaxel are plant-derived drugs used to treat cervical cancer clinically, alone, and as neoadjuvant chemotherapy with other chemotherapeutics such as cisplatin [5,6,7,8,9]; natural products are essential molecules since they comprise 50% of anti-tumor drugs [10,11]. Iridoids, a diverse class of secondary metabolites, are present in numerous plant families, with a prevalent occurrence in Apocynaceae, Loganiaceae, Scrophulariaceae, and others [12,13]. Across cultures and civilizations, iridoid-rich plants have been employed for their medicinal properties [12,13].

Structurally, iridoids are atypical monoterpenoids and are generally described as cyclopentane [C]-pyran. The cyclopentane is fused to a six-member oxygenated ring, forming the iridoid skeleton as a bicycle system [14,15]. The cyclopentane is fused to a six-member oxygenated ring, forming the iridoid skeleton as a bicycle system [14]. Over 2500 known iridoids are derived from nature, distinguished by the iridoid skeleton’s type and number of substituents [15]. The present work is related to two principal groups: simple iridoids and iridoid glycosides. Simple iridoids are iridoids that have a simple modification of the cyclopentane ring, and such modifications could be hydroxyl, acyloxy, keto, epoxy, chlorine, and olefins. Iridoid glycosides comprise a cyclopentane ring linked to a dihydropyran ring, with a sugar moiety commonly in R1; mainly, they are β-D-glucosides, and could have many different substituents in the sugar moiety such as coumaroyl, feruloyl, caffeoyl, and cinnamoyl groups [13] (Figure S1). Iridoid glycosides are sources of lead molecules as they share high modifiability and rapid absorption [12].

Iridoids have demonstrated significant bioactivity, with a growing body of research suggesting their potential in the prevention and treatment of cancer [12,16,17]. These compounds exhibit a range of pharmacological effects, including anti-inflammatory, antioxidant, and anti-proliferative activities, making them intriguing candidates for anticancer interventions. Studies have delved into the specific mechanisms through which iridoids exert their anticancer effects. In vitro studies about Geniposide and Genipin as potential compounds against different types of cancer cells have been reported [18,19,20,21,22,23,24,25]. Iridoids appear to target various hallmarks of cancer; these include inhibiting uncontrolled cell growth, promoting apoptosis, and impeding angiogenesis. The ability of iridoids to modulate multiple pathways involved in cancer development and progression makes them attractive candidates for further investigation [17]. The exploration of iridoids as anticancer agents represents a dynamic and evolving field within cancer research. While challenges exist, the multifaceted pharmacological activities of iridoids make them promising candidates for further investigation and potential development into novel cancer therapeutics.

Molecular similarity analysis is important in drug discovery because it helps to identify new drug candidates based on the structural and/or functional similarity of other drugs [26]. Moreover, ligand-based drug design is a method that combines mathematical modeling, and the physicochemical and structural properties, of a variety of biologically active chemical structures, to find the key elements that trigger their biological activity. This information is employed for the discovery of new structurally different drug candidates or for the optimization of lead compounds. Quantitative structure activity relationships (QSAR) in cancer are a powerful tool that considers strict statistical parameters to generate an equation or an algorithm that describes the relationship between the biological activity and one or more properties of the compounds [27].

The aim of the present work was to evaluate Geniposide and Genipin against different cervical cancer cells. From the obtained results, a molecular similarity analysis was undertaken using several active iridoids to explain the biological activity of Geniposide and Genipin. This molecular similarity analysis was undertaken by comparing the electronic properties (molecular potential maps and dipole moment) of all the iridoids. We found that the dipole moment is crucial for the biological activity of simple iridoids. Finally, after generating a QSAR model, we carried out a ligand-based drug design of new simple iridoids with potential cytotoxic activity.

## 2. Results and Discussion

### 2.1. In Vitro Cytotoxic Activity of Geniposide and Genipin

Table 1 shows the IC_50_ values of Geniposide and Genipin in three cervical cancer cell lines. Geniposide exhibited no activity in these cell lines. Genipin exerted a similar effect in the CaSki and CaLo cell lines and exhibited less potency in the INBL cell line. Although these cancer cell lines are from the cervix, they exhibit different molecular characteristics. CaSki is associated with HPV16 and derived from metastasis, while both CaLo and INBL are associated with HPV18. However, CaLo represents an early stage (stage IIB), while INBL represents a metastatic stage (stage IVB) according to the FIGO classification [28]. This suggests that Genipin could potentially affect cervical cancer cells from both high-risk HPV types (16 and 18) and from different stages of the disease.

In the literature, there is a vast number of glycosylated iridoids against various types of cancer in vitro. However, in this study, Geniposide did not exert any cytotoxic effects in any of the cell lines tested. On the contrary, Genipin emerged as an active compound against all the tested cell lines. Similar findings regarding the effects of Geniposide and its aglycone, Genipin, have been reported in relation to other types of cancer cell lines: *MCF-7 breast*, *HT-29 colon*, *K562 leukemia*, *U251 glioma*, *786-0 renal*, and *PC-3 prostate* [29]. Some iridoid glycosides do not inherently possess antiproliferative activity; instead, they exhibit effects on human cancer cells once they undergo hydrolysis [30]. Our objective was to investigate the relationship between the chemical structure, molecular features, and the cytotoxic activity of both iridoids.

### 2.2. Cytotoxic Iridoids Assessed against the HeLa Cell Line

To understand the lack of activity of Geniposide and the low activity of Genipin, both of which were tested against cervical cancer cells, we conducted a search for reported iridoids with cytotoxic effects against cervical cancer in vitro. We identified forty-four iridoids (Figure S2), primarily evaluated in cervical cancer cell lines, including HeLa, KB-3-1, and HeLa-S3, with exposure times of 24, 48, and 72 h [31,32,33,34,35,36,37,38,39,40,41,42,43]. Table 2 lists the 13 iridoids that were selected because they were assessed in the same cell line (HeLa) and for the same treatment duration (48 h). Additionally, these iridoids exhibited structural similarities to Geniposide and Genipin, with minor variations in their substituents over the core of the iridoid skeleton; iridoids with greater modification on the sugar core were not considered.

As there was no reported cytotoxic activity of Geniposide and Genipin against the HeLa cell line in the literature, as indicated by its IC_50_ at 48 h [34], we conducted our own test using the MTS method. The IC_50_ for Genipin at this time of treatment was found to be 419 ± 27.25 µM, with paclitaxel used as the positive control (IC_50_: 15.8 ± 0.67 nM).

**Figure 1:**
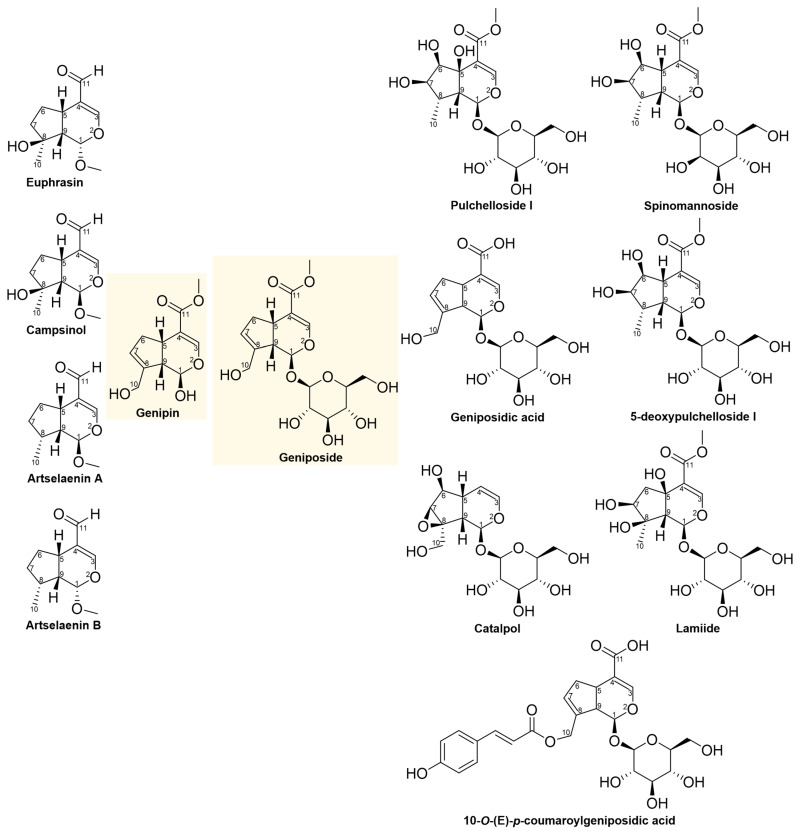
Chemical structures of iridoids tested against the HeLa cell line; Genipin and Geniposide are the central iridoids in this study marked in yellow color.

The structures of the 13 iridoids listed in Table 2 are depicted in Figure 1. Among these, five iridoids are simple (non-glycosylated, including Genipin), and eight iridoids are glycosylated (including Geniposide). All these iridoids were tested against the HeLa cell line under the same treatment condition of 48 h. The chemical structures of these iridoids typically featured substituents such as: methyl, hydroxyl, hydroxymethyl, ester, acid, aldehyde, and glucose. In some specific cases, mannose, epoxy, and *p*-coumaroyl substituents were also present. The iridoid skeleton (as shown in Figure S1) in certain instances exhibited a double bond between C7 and C8, like Genipin and geniposide.

Subsequently, to obtain more precise geometry and energy values of the molecules, a reoptimization process was undertaken using the density functional theory (DFT) approach. The objective was to analyze the structure–activity relationship of the iridoids (Figure 1) based on their electronic distribution and derived properties (polarizability, hardness, molecular electrostatic potential, and dipole moment).

### 2.3. Structural and Molecular Analysis of Genipin and Other Simple Iridoids

As an initial step, Euphrasin, Campsinol, Artselaenin A, and Artselaenin B [31] were compared in terms of their structure, activity, and molecular descriptors against Genipin. Table 3 presents the IC_50_ experimental values, and molecular descriptors calculated with Spartan’20, for the lowest-energy conformer of each simple iridoid, such as dipole moment, area, volume, polar surface area (PSA), ovality, LogP, polarizability, and the number of hydrogen bond donors (HBD) and acceptors (HBA). The first four simple iridoids demonstrated the highest potency, with their IC_50_ values being less than 14 µM, compared to Genipin. Genipin, in contrast, was 30- to 2000-fold less active. Artselaenin A had a GAP_HOMO-LUMO_ value more like that of Genipin. However, they differ in terms of polarity, as evidenced by the polar surface area values. Genipin had a higher value for this descriptor (64.589 Å^2^) compared to the others (ranging from 26.808 to 46.922 Å^2^).

The dipole moment of the remaining simple iridoids was higher, ranging from 4.2 to 5.78 debye, compared to Genipin, which exhibited a lower dipole moment of 1.26 debye. The dipole moment is a measure of the polarity of a molecule, established when there is an unequal distribution of electrons due to differences in electronegativity within the molecule. Consequently, the degree of electron distribution inequality is greater in simple iridoids than in Genipin, which appears to favor their biological activity. Additionally, Euphrasin and Campsinol exhibited hydrophilic properties like those of Genipin, while Artselaenin A and B displayed greater hydrophobicity than the others. Genipin also featured more HBD and HBA. The first iridoids closely resembled Genipin in terms of size (area and volume) and shape, as evidenced by their ovality values (ranging from 1.34 to 1.36). Furthermore, they shared similar polarizability values (ranging from 57.01 to 57.91).

**Figure 2:**
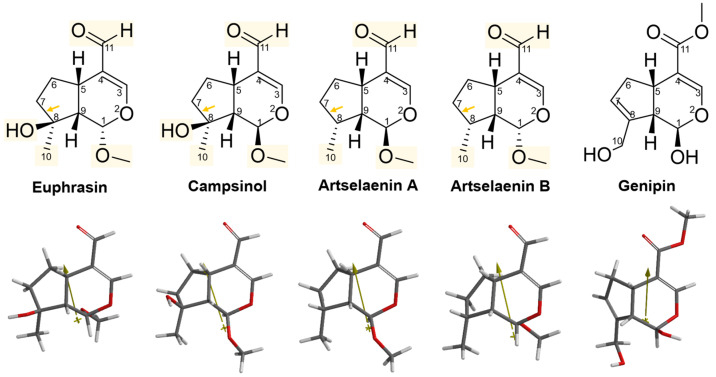
The 2D and 3D structures of simple iridoids including Genipin. The yellow rectangles indicate the change or addition of some substituents with respect to Genipin, the arrows indicate the lack of the double bond in cyclopentane respect to Genipin. The dipole vector is shown as a gold arrow over the 3D structures.

The 2D and 3D structures of simple iridoids are presented in Figure 2. Genipin has several structural differences compared to the other simple iridoids: it has a carbomethoxy group at C11, while the others have an aldehyde group. It has a double bond between C7 and C8 and the others lack this structural feature. In addition, it has a hydroxyl group at C10 instead of a hydroxyl group at C8. Genipin presents a free hydroxyl at C1, while in the other iridoids there is a methoxy group. Furthermore, the dipole angle in Genipin differs from that of Euphrasin, Campsinol, Artselaenin A, and Artselaenin B (Figure 2).

**Figure 3:**
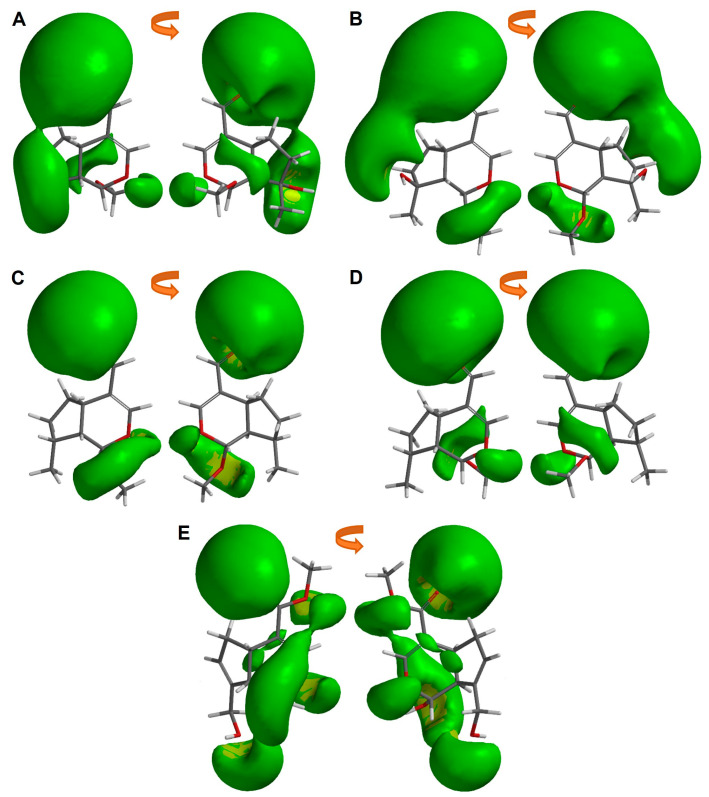
Molecular electrostatic potential iso-surface of −10 kcal/mol of simple iridoids. (**A**) Euphrasin, (**B**) Campsinol, (**C**) Artselaenin A, (**D**) Artselaenin B, (**E**) Genipin.

After analyzing the dipole orientation and magnitude, our interest turned to the electrostatic potential distribution of each iridoid. For that reason, an iso-surface of −10 kcal/mol value of MEP was obtained (see Figure 3). It is noteworthy that the most cytotoxic iridoids (Figure 3A,B) exhibit a higher electrostatic distribution in the aldehydic substituent at C4, which also features a hydroxyl at C8. Genipin, on the other hand, shows a divided electrostatic distribution on its substituent in C4 (partially over the sp2 and sp3 oxygens) and a greater electrostatic distribution in the pyran ring, along with the hydroxyl at C1 and C10. These observations revealed specific regions where the simple iridoids could interact with positively charged species at an energy level of −10 kcal/mol. The higher electron density over the aldehydic group and the hydroxyl group in C8 allows a better interaction with the possible target of these iridoids.

**Figure 4:**
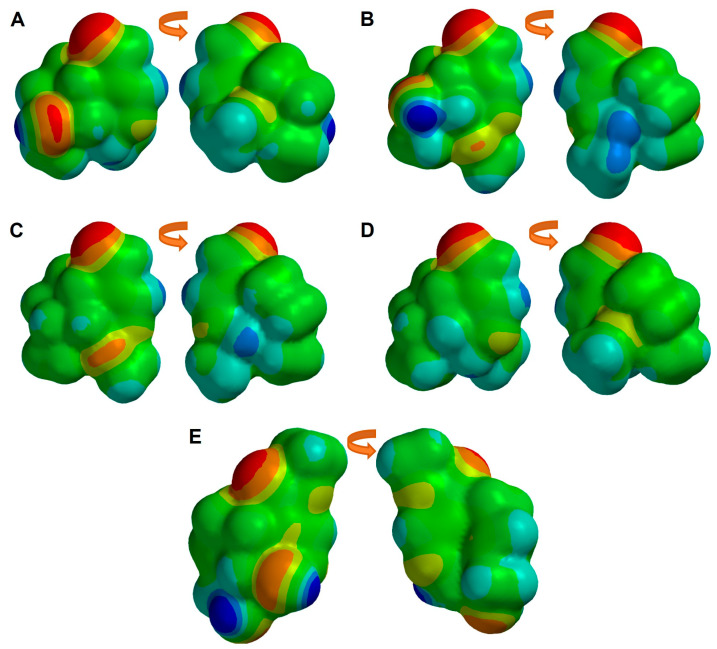
Molecular electrostatic potential map of simple iridoids: (**A**) Euphrasin, (**B**) Campsinol, (**C**) Artselaenin A, (**D**) Artselaenin B, (**E**) Genipin. Zero, negative, and positive values of MEP are depicted as green, red, and blue colored regions, respectively.

Then, the MEP map was obtained (Figure 4), indicating the regions more susceptible to an electrophilic attack (yellow to red color) and those more susceptible to a nucleophilic attack (blue color). The regions with more electronic density are located at the oxygen of the aldehydic group at C4, and the oxygen of the hydroxyl at C8. In the case of Genipin, these zones of higher electron density also correspond to the sp2 and sp3 oxygens, the oxygen of the hydroxyl at C1, and the oxygen of the hydroxyl at C10, correlating with our earlier observations regarding molecular electrostatic distributions.

These molecular descriptors and structural features affect the reactivity of the simple iridoids, particularly the substituent at C4 (aldehyde or ester), added to the forward hydroxyl at C8. Now, we are interested in studying the physicochemical differences between the conformer of lower energy of Geniposide and Genipin.

### 2.4. Structural and Molecular Analysis of Genipin and Geniposide

Genipin and Geniposide descriptors are presented in Table 4. Geniposide was inactive against cervical cancer cells, but Genipin was cytotoxic against the HeLa cell line. Both iridoids differ in the GAP_HOMO-LUMO_, LogP, and dipole moment. Due to the presence of a glucose unit in Geniposide, the size and the volume increased considerably compared to the Genipin 132 and 136 units, respectively. Also, the molecular shape is different between them, as we can see from the ovality parameter (1.36 for Genipin and 1.53 for Geniposide). The polarity of Geniposide was 2.5-fold higher than Genipin, based on the molecular charge and the polar surface area.

**Figure 5:**
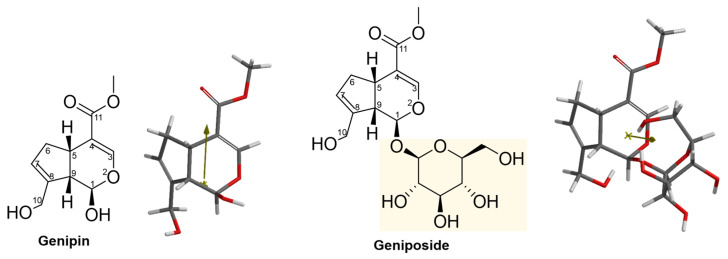
The 2D and 3D structures of Genipin and Geniposide. Yellow rectangle indicates the addition of the glucose with respect to Genipin. The dipole vector is shown as a gold arrow over the 3D structures.

Moreover, the Genipin and Geniposide 2D and 3D structures are displayed in Figure 5. The only structural difference between them is the glucose moiety at C1. We can observe the contrary direction of the vector dipole, that in Genipin is directed to the carbonyl at C11, and in Geniposide is directed to the glucose moiety, specifically to two hydroxyl groups in the sugar.

**Figure 6:**
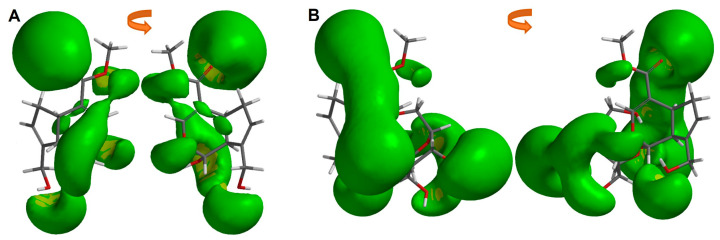
Molecular electrostatic potential iso-surface of −10 kcal/mol of (**A**) Genipin, (**B**) Geniposide.

After analyzing the dipole orientation and magnitude, we obtained the molecular electrostatic potential iso-surface of −10 kcal/mol showing the electrostatic profile of both iridoids (Figure 6). A higher electronic distribution in the carbonyl at C11 until the 3′ and 6′ hydroxyls of the sugar is observed in Geniposide, while in Genipin a different pattern is presented. In these green zones, intermolecular interactions could take place, such as hydrogen bonds or electrostatic interactions with a positively charged group.

**Figure 7:**
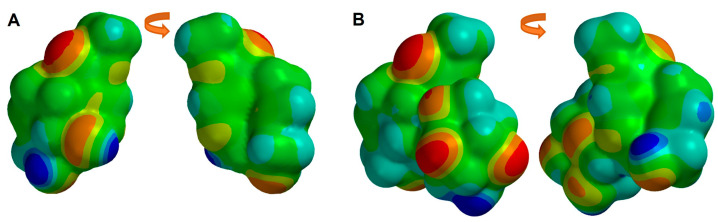
Molecular electrostatic potential map of (**A**) Genipin, (**B**) Geniposide. Zero, negative, and positive values of MEP are depicted as green, red, and blue colored regions, respectively.

The regions with more electron density of both iridoids are displayed in Figure 7. In Geniposide, these regions are located mainly at the oxygen of the carbonyl at C11, the oxygen of the hydroxyl at C10, and the 3′, 4′, and 6′ hydroxyls in the sugar. In Genipin these zones of more electron density also correspond to the carbonyl at C11, the sp3 oxygen of the ester at C11, the oxygen of the hydroxyl at C1, and the hydroxyl at C10.

**Figure 8:**
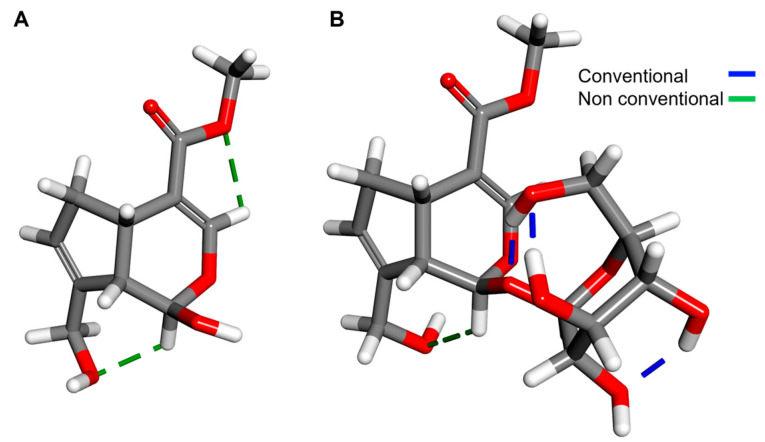
Intramolecular H bonds in (**A**) Genipin, (**B**) Geniposide.

Finally, intramolecular hydrogen bonds are observed in Figure 8. These intramolecular interactions were displayed and obtained with the Discovery Studio 2021 software [44]. In Genipin, two non-conventional hydrogen bonds are present, while in Geniposide there is one non-conventional and three conventional hydrogen bonds. Sugar conformation in Geniposide causes the formation of these types of strong interactions that confer stability to the iridoid.

The increased polarity of Geniposide due to the incorporation of the glucose in C1, and the distribution of this molecular charge, impact on the electronic distribution, and finally on the inactivity of the iridoid, unlike Genipin. Now, our interest is turned to comparing the physicochemical differences between Geniposide and active iridoid glycosides.

### 2.5. Structural and Molecular Analysis of Geniposide and Other Iridoid Glycosides

In Table 5 are listed some descriptors related to the reactivity, solubility, shape, and size of active iridoid glycosides and Geniposide. The iridoid glycosides Pulchelloside I, Catalpol, Lamiide, Spinomannoside, 5-deoxypulchelloside I, geniposidic acid, and 10-O-(E)-p-coumaroylgeniposidic acid have a similar cytotoxic activity against the HeLa cell line (25.22–48.10 µM). However, compared to the inactive Geniposide, all of them are different in size, and in HBD and HBA. Conversely, Geniposide was similar in form, as given by the ovality parameter, and in the polarizability, but it was quite different in the molecular charge distribution given by the dipole moment, and it was less polar compared to the rest of the iridoids (less hydrophilic). The anion forms of geniposidic acid and 10-O-(E)-p-coumaroylgeniposidic acid were considered since these charged forms exist predominantly at physiologic pH (7.4). The pKa value of the acids are shown in Table S2.

**Figure 9:**
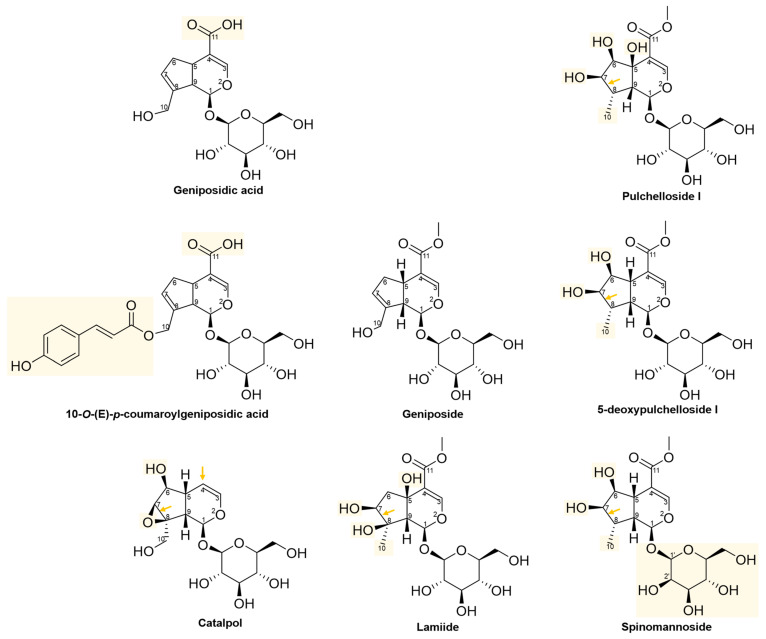
The 2D chemical structures of iridoid glycosides. The yellow rectangles indicate the change or addition of some substituents with respect to Geniposide, the arrows indicate the lack of the double bond in cyclopentane, and the lack of the substituent at C4 with respect to Geniposide.

The 2D structures of iridoid glycosides are presented in Figure 9. These iridoids have a glucose moiety at C1, except Spinomannoside, which contains a mannosyl at C1 instead of the glucosyl. The 5-deoxypulchelloside has two extras’ hydroxyls at C6 and C7, lacks the double bond in C7–C8, and has a methyl instead of the hydroxymethyl at C8. Pulchelloside I, in addition to these structural differences compared to Geniposide, also presents an extra hydroxyl at C5. Spinomannoside, which is structurally very similar to 5-deoxypulchelloside, contains a mannose at C1 instead of the glucose. Lamiide presents a hydroxyl at C5, C7, and C8, lacks the double bond in C7–C8, and has a methyl instead of the hydroxymethyl at C8. Catalpol presents an epoxy in C7–C8, lacks the double bond in C7–C8, contains a hydroxyl at C6, and lacks the ester at C4 compared to Geniposide. Geniposidic acid has an acid at C4, just like 10-*O*-(E)-*p*-coumaroylgeniposidic acid (this last one additionally presents a *p*-coumaroyl group at C10), contrary to Geniposide, which presents an ester at C4. These structural changes, compared to Geniposide, seem to confer cytotoxic activity against the HeLa cell line.

The 3D structures of the iridoid glycosides, showing the dipole vector, are displayed in Figure S3. The most similar to Geniposide is Catalpol, although the conformation of the sugar is very different compared to Geniposide. In Pulchelloside I and Spinomannoside, the conformation of the sugar was more like the one displayed in Geniposide. It is noteworthy how the conformation of the iridoids impacted on their size and shape, as is noted in the area, volume, and ovality values. For example, Pulchelloside I and Lamiide, although they have the same number and type of substituents, the conformation of the glucose increased the area in Lamiide (Figure S3).

Moreover, we were interested in visualizing the regions where the intermolecular interactions can occur. For this reason, an iso-surface of −10 kcal/mol value of MEP was obtained (Figure S4). It can be observed that the most cytotoxic glycosylated iridoid (Figure S4f) has a higher electrostatic distribution, principally on the region rich in hydroxyls on the cyclopentane, which are excellent acceptors of hydrogen bonds. This electrostatic distribution is also present on the oxygen of the carbonyl at C11 and is extended until the hydroxyl at C6. In the case of geniposidic acid (Figure S4a), a similar region and electrostatic distribution is present, principally due to the hydroxymethyl at C8. The rest of iridoid glycosides show several and similar electron density regions where the intermolecular interactions could occur.

In addition, we obtained the MEP map of the glycosylated iridoids (Figure S5). The regions with more electron density in Geniposide are located mainly at the oxygen of the carbonyl at C11, the oxygen of the hydroxyl at C10, and the 3′, 4′, and 6′ hydroxyls of the sugar. A greater number of susceptible regions to electrophilic and nucleophilic attacks are observed in glycosylated iridoids. Additionally, we performed molecular similarity analysis of the most cytotoxic iridoids and the central iridoids of this study.

### 2.6. Molecular Similarity Analysis of Iridoids

**Figure 10:**
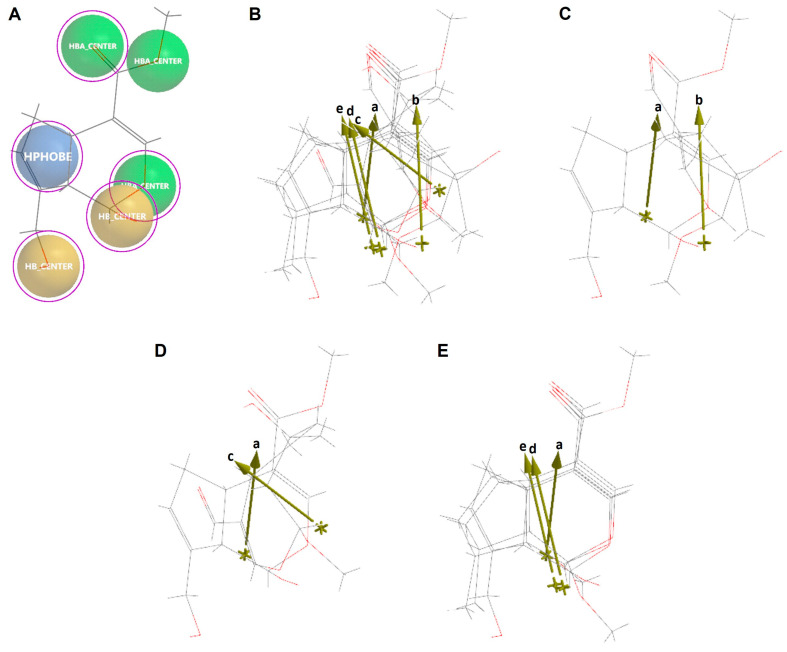
Alignment of Genipin with simple iridoids Euphrasin, Campsinol, Artselaenin A, and Artselaenin B. (**A**) Common similarity centers by CFDs of Genipin (purple circles represent the CFDs selected for the alignment). (**B**) Alignment of Genipin with simple iridoids. (**C**) Alignment of Artselaenin A with Euphrasin. (**D**) Alignment of Artselaenin A with Campsinol. (**E**) Alignment of Artselaenin A with Artselaenin A and Artselaenin B. Dipole vector represented by gold arrows of each iridoid aligned is shown (a: Genipin, b: Euphrasin, c: Campsinol, d: Artselaenin A, e: Artselaenin B).

Molecular similarity analysis of the simple iridoids with Genipin was performed based on the chemical function descriptors (CFDs). The alignment and the dipole vector of the five iridoids are presented in Figure 10. Five CFDs were selected in Genipin as they were common with the other four simple iridoids (Euphrasin, Campsinol, Artselaenin A, and Artselaenin B). The most active iridoids, Euphrasin and Campsinol, did not align well with Genipin (Figure 10C,D); their alignment scores were 0.50 and 0.49, respectively. Otherwise, Artselaenin A and Artselaenin B, in which the only difference is the stereochemistry of the methoxy group at C1, had parallel dipole vectors that present a different orientation to the dipole vector of Genipin (Figure 10E); their alignment scores were 0.56 and 0.50, respectively.

Additionally, to verify the difference in the chemical natures between Geniposide and its aglycone Genipin, we performed a molecular similarity analysis by the CFD alignment that is shown in Figure S6. Six CFDs were selected in Genipin as they were also common to Geniposide; the alignment score was 0.65. Although Geniposide and Genipin share those selected CFDs, it is evident that there is a difference in their orientation and the direction of the dipole vector, which could explain the lack of biological activity of Geniposide (Figure S6).

### 2.7. Ligand-Based Drug Design of Genipin Derivatives

Afterward, we were interested in designing iridoids based on Genipin’s structure. We noticed that the dipole vector correlated with the biological activity of the most cytotoxic iridoids (Table 3). Therefore, we obtained the dimensional components of the dipole vector of these simple iridoids. The Cartesian coordinates of the dipole vector were transformed to polar spherical coordinates and their values are presented in Table 6, where ρ corresponds to the dipole moment magnitude, θ is the angle in the polar coordinates, and φ is the azimuthal angle.

Derived from this set of data, a multilinear regression analysis considering the azimuthal angle (*φ*), the dipole moment magnitude (ρ), and the potency of the iridoids (${-\log IC}_{50}$), was performed and we obtained a mathematical model that describes the biological activity of simple iridoids:

(1) $${-\mathrm{Log}IC}_{50}=1.5425\left(\varphi \right)+0.8941\left(\rho \right)-6.9922$$

$${\;R=0.89;\;R}^{2}=0.79;\;s=0.79;\;F=3.69;\;n=5$$

**Figure 11:**
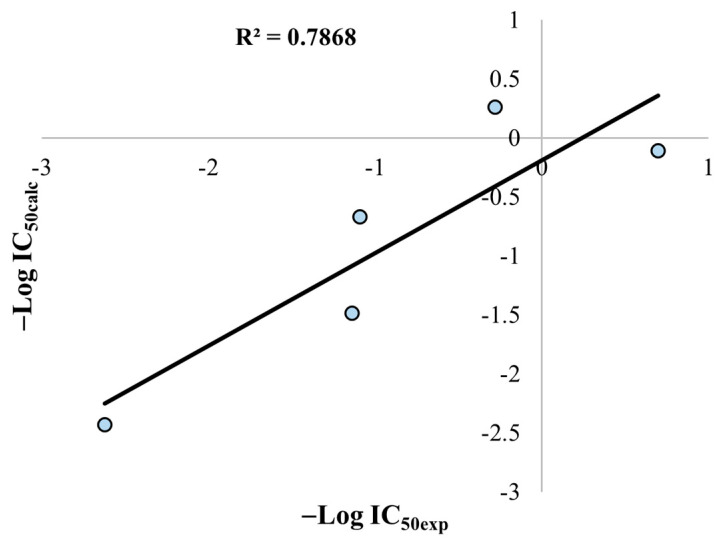
Linear correlation of the calculated activity (IC_50calc_) versus experimental activity (IC_50exp_).

According to this equation and its statistical parameters, the potency of simple iridoids will increase proportional to the magnitude of the dipole moment and the azimuthal angle *φ*. Then, we plotted the linear correlation between the calculated and the experimental activity, which is displayed in Figure 11. The squared correlation (*R*^2^), which explain the variance of the description model, is shown in the plot. The plot of Figure 11 shows that the mathematical model has an acceptable descriptive ability, considering the limited number of compounds employed for the model.

Derived from this analysis we observed that the dipole moment was crucial for the activity of the iridoids, but the model considering the magnitude and the angle φ of the dipole vector was only descriptive, and not predictive for other iridoids. For this reason, we considered all 13 iridoids to perform a QSAR study using molecular descriptors related to the electronic nature of the compounds. QSAR models of a small set of molecules have been successfully employed to help explain their biological activity.

### 2.8. QSAR Analysis

The final QSAR model indicates once again the influence of the dipole moment on the activity of the iridoids, as shown in Equation (2). Moreover, the difference of the polar surface area of the iridoids with respect to Genipin (∆PSA), the solubility of the iridoids in water (LogS), and the difference in the polarizability of the iridoids with respect to Genipin (∆Polarizability) are relevant for the biological activity.

(2) $$-\mathrm{Log}{IC}_{50}=0.1118\left(\rho \right)-0.07433\left(\Delta PSA\right)+2.56022(LogS)+0.02923\left({∆Polarizability}^{2}\right)+1.26184$$

$${\;R=93.781;\;R}^{2}=87.95;\;R_{ADJ}^{2}=81.07;\;s=0.3563\;F=12.78;\;n=13$$

$${\;R^{P}=0.\;622;\;R}^{N}=-0.208;\;Q_{LOO}^{2}=62.33$$

The values of the molecular descriptors considered in the QSAR model are listed in Table 7. In addition, all the experimental values of the cytotoxic activity against the HeLa cell line (IC_50_) are shown. From Table 7, it can be seen that the most potent iridoids (simple iridoids) have a lower PSA value than Genipin, since its difference (∆PSA) is negative. This agrees with our QSAR equation since the negative coefficient indicates that increasing the PSA value of the iridoids compared to Genipin will negatively affect its cytotoxic activity against the HeLa cell line. On the contrary, an increase in the solubility or the polarizability of the iridoids will favor the cytotoxic activity of iridoids according to their coefficient positive sign. These descriptors can work as counterweights of each other, specially ΔPSA and LogS. It is worth mentioning that all the iridoids used for the construction of the QSAR model have a LogS negative value, that reflects their hydrophobic nature. This feature affects the positive contributions of the dipole moment and the ΔPolarizability, descriptors related to the electron density distribution over the iridoid framework. Iridoids glycoside have higher values for the dipole moment, ΔPSA, and ΔPolarizability. Nevertheless, some of them have a lower LogS value than Genipin, and this can be related to their sugar moiety and its ability to form intramolecular hydrogen bonds, making the iridoid glycosides more hydrophobic.

All the experimental biological activities (−Log IC*_50_*_exp_ is represented as Y_exp_), the calculated and predicted biological activities (−Log IC_50calc_ is represented as Y_calc_ and −Log IC_50pred_ is represented as Y_pred_) by the QSAR model, and leverage values (Hat) of iridoids are presented in Table 8. Also, the calculation error and prediction error values that state the differences between Y_exp_ and both Y_calc_ and Y_pred_, are presented by the residual_calc_ and the residual_pred_ terms, respectively.

**Figure 12:**
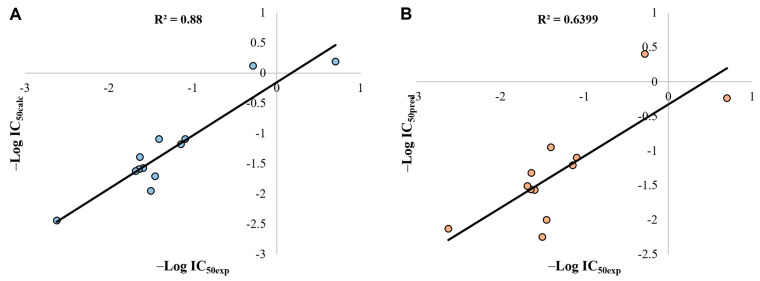
(**A**) Linear correlation of calculated activity (Y_calc_) versus experimental activity (Y_exp_). (**B**) Linear correlation of predicted activity (Y_pred_) versus experimental activity (Y_exp_).

Figure 12 displays the descriptive and predictive ability of the QSAR model, according to the squared regression coefficient (R^2^)–that corresponds to the value of the explained variance in the description and the prediction (Q^2^)–our model has an acceptable ability to predict biological activity. Campsinol and Lamiide were the outliers of the correlation prediction, since the polar surface area of Lamiide was higher than the PSA value for other similar iridoids, and consequently the difference with the PSA of Genipin did not permit a reasonable adjustment.

**Figure 13:**
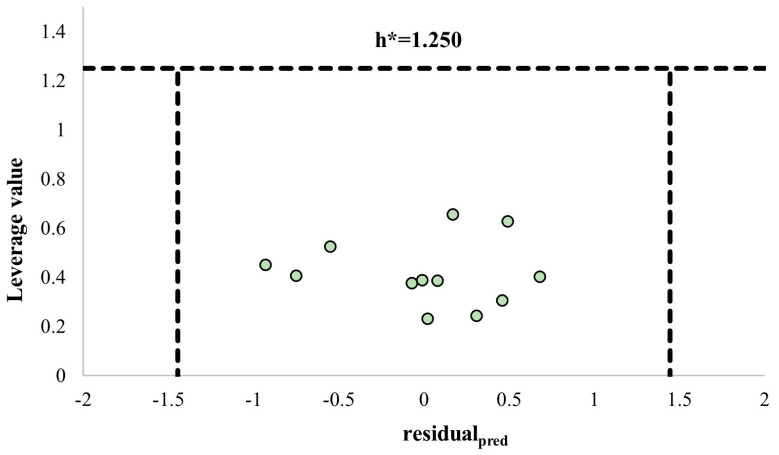
The William plot of prediction residuals versus leverage values of iridoids. The horizontal dashed line shows the warning leverage (h* = 3 p/n, n is the number of iridoids, and p is the number of descriptors in the model plus one), and the two vertical dashed lines indicate the limits within which all residuals should lie (3×SDEP = 1.444).

William’s plot, based on the prediction residuals and the leverage values, was used to define the applicability domain of the model (Figure 13). Structural (h > h*) and response outliers (residual_pred_ > 3SDEP) can be observed outside the area limited by the three dashed black lines. These lines represent the warning leverage (h*, dashed horizontal line), and three times the standard deviation in the prediction error (3×SDEP, dashed vertical lines). All the iridoids fell within the applicability domain of the model, suggesting the application of the model for predicting the cytotoxic activity of new iridoids with remarkably high molecular and structural similarity.

Now, we proceed to design iridoids based on the principal structural characteristics of Genipin. As Genipin is an active molecule with low potency, it could be considered as the lead compound in anticancer drug development.

**Figure 14:**
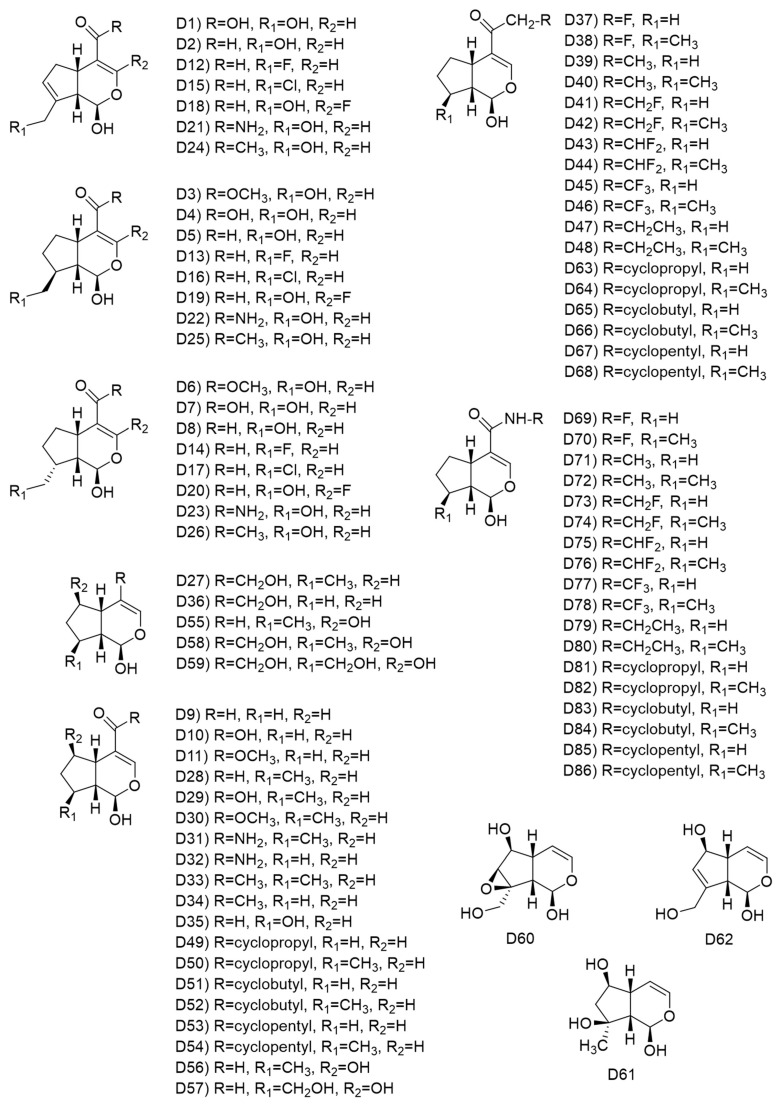
Chemical structures of designed simple iridoids based on Genipin.

The 2D structures of a set of iridoids based on the structure of Genipin, including some derived from glycosylated iridoids, are shown in Figure 14. The hydroxyl at C1 of Genipin was conserved in these proposed iridoids, due to its crucial role for the cytotoxic activity of Genipin derivatives, as reported by Yang et al. [45] for a pancreatic cancer cell line (Panc-1). Some structural elements of simple active iridoids (Euphrasin, Campsinol, Artselaenin A, and Artselaenin B) were considered. First, we decided to change the functional group of C4, as we saw its effect on simple and glycosylated iridoids in an increased biological activity, following this order: ester (Genipin), carboxylic acid (D1), and aldehyde (D2). We also considered primary amide (D21-D23) and ketone (D24-D26) as substituents of C4. Then, we evaluated the effect of the lack of the double bond in C7–C8 in the biological activity, plus the effect of the functional group present in C4, with both stereoisomers of the hydroxymethyl at C8 (see Figure 14). We also evaluated the introduction of fluorine in position 3 of the iridoid skeleton in three cases. For those Genipin derivatives we also modified the substituent of C10, represented by R_1_. These Genipin derivatives are comprised from D1-D8, and D12-D26. The rest of the iridoids were based on the simple iridoids reported in the literature, some present in iridoid biosynthetic pathways [46,47], such as 7-deoxyloganetic alcohol (D27, also named 7-deoxyloganetol), 7-deoxyloganetic aldehyde (D28, also named iridotrial), 7-deoxyloganetic acid (D29), and 7-deoxyloganetin (D30). These iridoids include D9-D11, D27-D59, D63-D90, with diverse structural modifications in C4, conserving the carbonyl and adding methyl groups over the cyclopentyl. Finally, we include a third set of three iridoids, which are derived from glycosylated iridoids (D60-D62), that have shown inhibitory activity against Taq DNA polymerase [48].

The molecular descriptors of all the 86 designed iridoids were calculated and are presented in Table S3. We applied the QSAR model for the designed iridoids with these values and predicted the biological activity (IC_50pred_) for all the compounds. The results for the best candidates are presented in Table 9. Finally, the best results are: 0.053, 0.002, 0.006, 0.011, 0.146, 0.103, 0.011, 0.029, and 0.021 µM for D9, D10, D35, D36, D55, D56, D58, D60, D61, and D62, respectively.

**Figure 15:**
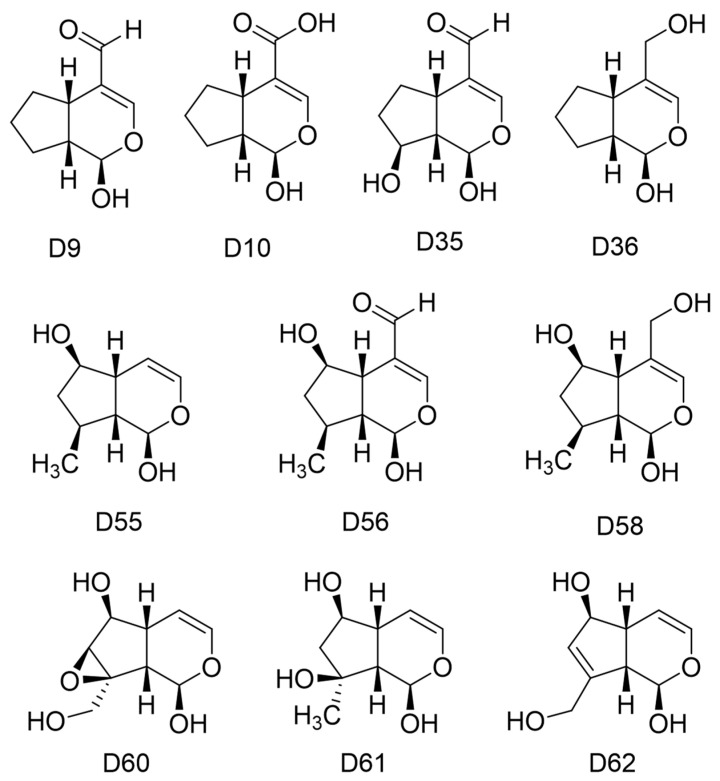
Chemical structures of the best designed iridoids based on Genipin.

After applying the QSAR model of Equation (2), a set of ten iridoids had a higher predicted biological activity than the experimental activity of Euphrasin (Figure 15), IC_50pred_ lower than 0.2 µM. Most of these designed iridoids had an aldehyde or an hydroxymethyl in C4, but four of them lacked a substituent in this position. Only one of them had an acid in this position D10. All of them conserved the hydroxyl in C1, and others featured a methyl or a hydroxyl in C8, or both, and a hydroxyl in C6. Only two iridoids, D60 and D62, presented an hydroxymethyl in C8, as with Genipin, but D60 had an epoxy group between C7 and C8. D62 was the only iridoid with a double bond in C7–C8, as with Genipin.

In Table 9, the descriptors of these ten designed ligands are presented. The ligands with an aldehyde in C4 (D9, D35, and D56) had similar GAP_HOMO-LUMO_ to Genipin; those having an acid or an hydroxymethyl, or no substituent in C4, had a less negative GAP_HOMO-LUMO_. D9, D10, and D56 had the highest values of dipole moment, but D36 and D62 had lower values of the dipole moment than that of Genipin (see Table 3). D9 and D10 were smaller in area and volume than the most cytotoxic iridoids. D60, D61, and D62 were more polar than Genipin, and the rest of the iridoids were less polar compounds compared to Genipin, with PSA values in the range 42–61 Å^2^. All the proposed iridoids had a higher affinity for water, except D36 and D55, which present a higher affinity for a lipidic phase, as with Artselaenin A and Artselaenin B. Almost all compounds were *less* polarizable molecules than Genipin and the other simple iridoids presented in Table 3. These iridoids had a variable number of hydrogen bond donors and acceptors, ranging from 1 to 4, and 3 to 5, respectively.

The 3D structures of the *best* designed iridoids based on the structure of Genipin, with their respective dipole vectors, are displayed in Figure S7. Curiously, only D9, D10, D36, and D56 present a dipole vector with a similar direction to the most active simple iridoids (Euphrasin, Campsinol, Artselaenin A, and Artselaenin B); this could be attributed to the aldehyde in C4, that was common to these four designed iridoids. The rest of the iridoids presented a dipole vector with a different orientation. This was influenced by the presence of other substituents in C4, or the lack of anyone.

It is noteworthy that the change of the ester for the carboxylic acid at C4 increased the potency of the iridoid compared to Genipin, but the change of the carboxylic acid by an aldehyde decreased the potency of the iridoid compared to the IC_50exp_ of Genipin. The elimination of the double bond in C7–C8 also increased the potency of the iridoids. In addition to the lack of the double bond in C7–C8 of Genipin, the absence of any substituent in the cyclopentane ring increased the biological activity, and the incorporation of a hydroxyl in C8, or an extra hydroxyl in C6 (D60-D62) increased the potency of the compound. In some cases, the reactivity order of the compound related to the substitutions in C4 is as follows: any > hydroxymethyl > aldehyde (D55, D56, and D58).

**Figure 16:**
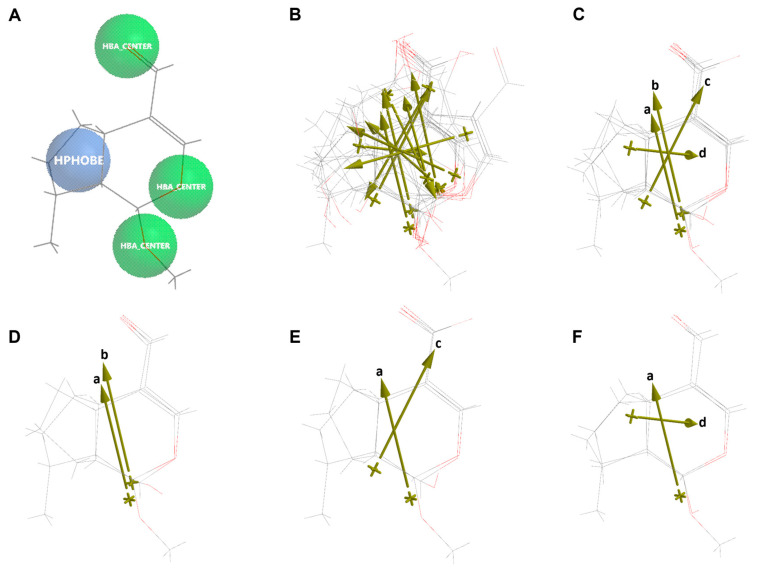
Alignment of Artselaenin A with the best designed iridoids. (**A**) Common similarity centers by CFDs of Artselaenin A (purple circles represent the CFDs selected for the alignment). (**B**) Alignment of Artselaenin A with D9, D10, D35, D36, D55, D56, D58, D60, D61, and D62. (**C**) Alignment of Artselaenin A with D9, D10, D36. (**D**) Alignment of Artselaenin A with D9. (**E**) Alignment of Artselaenin A with D10. (**F**) Alignment of Artselaenin A with D36. Dipole vector represented by gold arrows for each iridoid aligned is shown (a: Artselaenin A, b: D9, c: D10, d: D36).

Next, the alignment of the three best designed iridoids (D9, D10, D35, D36, D55, D56, D58, D60, D61, and D62) and the most structurally similar active iridoid (Artselaenin A) is shown in Figure 16. All four CFDs were selected since they are in common with the designed iridoids and Artselaenin A. The highest alignment scores were those between the designed iridoids D9, D10, and D36 with Artselaenin A, and their values were 0.78, 0.77, and 0.99, respectively. The best alignment score was obtained with D36, comparing the structure of the iridoid skeleton, but its dipole vector was not parallel to that of Artselaenin A (Figure 16F). The alignment with D9 had a lowest score of 0.78, but the orientation of the dipole vector was parallel to that of Artselaenin A (Figure 16D). These iridoids have not been evaluated against cervical cancer cells to our knowledge.

Finally, we analyzed the MEP graphics of the best candidates of the designed iridoids to evaluate the principal differences and similarities to explain their reactivity. The MEP isosurface of −10 kcal/mol shows the electrostatic profile of the designed iridoids (Figure S8). The increased calculated activity of the designed iridoids could be attributed to the higher electron density over the carboxylic acid or aldehyde at C4, the hydroxyl at C1, and the sp3 oxygen in the pyran. Also, this higher electron density is observed on the oxygen of the hydroxymethyl in C4, and on the hydroxyls in C6, and C8. This electron density pattern is observed in Artselaenin A, which was the most similar to D9, D35, and D56 (Figure S8A,C,F). D61 and D62 also present a higher electron density over the hydroxymethyl at C8 (Figure S8I,J).

In Figure S9, regions most susceptible to an electrophilic attack (yellow to red color) and those to a nucleophilic attack (blue color) are displayed. In all iridoids the regions with more electronic density are located at the oxygen of the carbonyl at C4, and the oxygen of the hydroxyl at C1, C6, C8, and C10, correlating with the previous MEP distributions. The regions with less electron density were located principally over the hydrogen of the aldehyde at C4, and the other hydrogens of the hydroxyls at C1 and C10, which correlated to the other surface graphics, and were like the regions of the MEP map of Artselaenin A, principally D9, D35, and D56 (this last with an extra electron distribution on the sp2 oxygen). Iridoids such as D60 and D61 presented a greater electron distribution due to the hydroxyls present on their structures, and in the case of D60, due to the presence of the epoxy group.

## 3. Materials and Methods

### 3.1. In Vitro Cytotoxic Activity of Geniposide and Genipin

In vitro cytotoxicity activity of Geniposide and Genipin was measured using the sulforhodamine B (SRB) (MP Biomedicals, Irvine, CA, USA) protein staining assay [49,50,51], using three different cervical cancer cell lines CaSki, CaLo, and INBL; and the immortalized keratinocytes HaCaT, as a non-tumorigenic cell line. The CaLo and INBL cell lines [28] were donated by Profesor María de Lourdes Gutiérrez Xicoténcatl from Centro de Investigación sobre Enfermedades Infecciosas, Instituto Nacional de Salud Pública. Briefly, cells were cultured in RPMI-1640 medium supplemented with 10% fetal bovine serum, and seeded in a 96-well microtiter plate at a cellular density of 5000 cells/mL, and placed in an incubator (5% CO_2_ and 37 °C) for 5 h. Afterward, different concentrations of pure iridoids (0.04128, 0.2064, 1.032, 5.16, 25.8, and 129 µM), of podophyllotoxin positive control (0.00303, 0.00606, 0.01213, 0.02425, 0.04850, and 0.09700 µM), and of cisplatin positive control (0.10400, 0.20781, 0.41563, 0.83125, 1.6625, and 3.325 µM) were added in triplicate and incubated for 72 h. The assays were conducted in three independent experiments.

Afterward, cells were fixed with cold trichloroacetic acid (30% in water) and stained with SRB (0.4% in a 1% of acetic acid solution). Cells were washed with a cold 1% acetic acid solution. Finally, the bound colorant was solubilized with Tris base to obtain the optical density (ODsample). The bound colorant was proportional to either total protein or cells amount. DMSO (final concentration of 0.5%) was used as vehicle and blank (ODblank) in Genipin experiments, and only deionized water was employed as vehicle and blank in Geniposide experiments. The total protein concentration in a single plate with cells at the assay’s beginning was considered zero (ODzero). Microtiter plates were incubated for 72 h, after which the total protein concentration was determined with Equation (3). Using an ELISA-Reader spectrophotometer, this assay measures the respective absorbance at 490 nm (Molecular Devices, SPECTRA max plus 384). Results were expressed as the concentration that inhibits 50% of control growth after the treatment period (IC_50_), using the statistical program GraphPad Prism, version 8.00 (GraphPad Software, Inc, La Jolla, CA, USA).

(3) $$\%Survival=\frac{{OD}_{sample}-{OD}_{zero}}{{OD}_{blank}-{OD}_{zero}}\times 100$$

Cytotoxic activity assays with Geniposide and Genipin against the HeLa cell line were performed by the MTS method [52]. For this assay, 5000 cells were seeded in a 96-well cell culture plate and treated for 48 h using 882, 441, 220.50, 110.25, 55.125, 27.5625, and 13.78125 µM of Genipin or Geniposide, and 50, 25, 12.5, 6, and 3 nM of paclitaxel as positive control. DMSO (final concentration of 0.5%) was used as a vehicle and blank. We used CellTiter 96^®^ Aqueous One Solution Cell Proliferation Assay kit (Promega, Madison, WI, USA) to determine the number of viable cells, following the manufacturer’s instruction. Cell viability was estimated by measuring the absorbance at 450 nm using an automated ELISA reader (Promega, Madison, WI, USA). Data were analyzed in the Prism 5.0 statistical program (GraphPad Software, Inc, La Jolla, CA, USA), and the IC_50_ values were determined by regression analysis.

### 3.2. Searching of Cytotoxic Reports of Iridoids in Cervical Cancer

The information about the cytotoxicity of iridoids in cervical cancer cells in vitro was collected by searching on databases and websites such as PubMed, SciFinder, Google Scholar, Elsevier, ScienceDirect, and Web of Science. The following words or phrases were used in diverse combinations for searching: “cytotoxic iridoids in cervical cancer”, “iridoids against cervical cancer”, “antiproliferative iridoids in cervical cancer”, “cervical cancer”, “in vitro”, “cytotoxic iridoids”, “cell death”. More than 30 scientific works of literature were consulted from 2004 to the date, gathering forty-four iridoids with reported cytotoxic activity in a cervical cancer cell line.

Eleven of the forty-four iridoids were selected to analyze their similarity on their structure, molecular descriptors, and the reported cytotoxic activities to understand the absence of Geniposide activity and the low Genipin activity. The iridoids were selected based on the two following criteria: (1) they were assessed in the same cell line, and (2) they were evaluated at the same time of treatment.

### 3.3. Conformation Analysis, Geometry Optimization, and Energy Calculation

A conformational analysis of all the simple and glycosides iridoids in their neutral and anionic form using the MMFF94 molecular mechanism model was performed [53]. The minimum energy conformer was submitted to a geometry optimization without symmetry constraints, employing the PM6 semiempirical method [54]. A harmonic frequency analysis was performed to ensure that the structure corresponds to a minimum on the potential energy surface. Furthermore, to acquire a more precise energy value and electronic density characteristics, a geometry reoptimization, at a density functional theory level, with the B3LYP hybrid functional [55] and the 6-31G* basis set [56], was performed. These systems were evaluated in water within the SM8 model [57].

Additionally, a single-point calculation using the B3LYP functional and 6-311 + G** basis set [58] in vacuum (in water for 10-*O*-(E)-*p*-coumaroylgeniposidic acid) was performed.

### 3.4. Molecular Descriptors

The following global chemical reactivity descriptors: Energy of the Highest Occupied Molecular Orbital (E_HOMO_), Energy of the Lowest Unoccupied Molecular Orbital (E_LUMO_), dipole moment (ρ), polarizability, polar surface area (PSA), Hydrogen Bond Donnor (HBD), and Hydrogen Bond Acceptor (HBA) counts, and some related to their size and form: area, volume, ovality, and their solubility: LogP (1-octanol/water calculation) were obtained from the quantum calculations performed with SPARTAN’20 program [59].

### 3.5. Molecular Representation of Electron Density Properties

The molecular electrostatic potential (MEP) mapped onto an iso-density surface (0.002 e-/Å^3^) for each compound was obtained. The MEP map provides a perception of the molecular size and the location of electron-rich and -deficient zones on a compound. In addition, to evaluate the electronic effect caused by the substituents in the iridoid skeleton, a molecular electrostatic potential surface with a value of −10 kcal/mol was obtained. All the molecular graphics were performed in the SPARTAN’20 program.

### 3.6. Molecular Similarity Analysis of Iridoids

Molecular similarity analysis was performed by comparing the skeleton base of the iridoids and detecting the substituents that are changed or added between them to the central iridoid of the comparison (Genipin or Geniposide). A molecular similarity analysis of three groups was performed: (1) Genipin with other simple iridoids, (2) Genipin with Geniposide, and (3) designed iridoids with one of the most cytotoxic iridoids.

### 3.7. QSAR Construction and Validation

#### Molecular Descriptors

The following global chemical reactivity descriptors were considered to construct the QSAR model: Energy of the Highest Occupied Molecular Orbital (E_HOMO_), Energy of the Lowest Unoccupied Molecular Orbital (E_LUMO_), chemical hardness (η) expressed by equation 4, electronegativity (χ) expressed by Equation (5), dipole moment (ρ), polarizability, Δpolarizability, polar surface area (PSA), ΔPSA, Hydrogen Bond Donnor (HBD), and Hydrogen Bond Acceptor (HBA) counts, and some related to their size and form: area, Δarea, volume, Δvolume, ovality, Δovality, and their solubility: LogP (1-octanol/water calculation), ΔLogP, and LogP^2^. All the Δ are the differences in the values between the descriptor of the iridoid in case and the descriptor of Genipin. All the calculations were performed in the SPARTAN’20 program. LogS (aqueous solubility calculation) was an extra descriptor and was obtained from Virtual Computational Chemistry Laboratory [60]. The energy corresponding to E_HOMO_ represents the ionization potential of the molecule and E_LUMO_ the corresponding electron affinity value, according to the Koopmans approximation.

(4) $$\eta =\frac{E_{HOMO}-E_{LUMO}}{2}$$

(5) $$\chi =\frac{E_{HOMO}+E_{LUMO}}{2}$$

### 3.8. Mathematical Model Generation

To construct our mathematical model, the multilinear regression by minimum quadratic differences was employed, using Excel Microsoft Office 365. All the molecular descriptors and the biological activity (IC_50_) of the iridoids were used as the independent variables (X) and the dependent variable (Y), respectively.

### 3.9. Statistical Validation

For the validation of our QSAR model, we employed the coefficient of determination (*R*^2^) (Equation (6)), cross-validated *R*^2^ (*Q*^2^), standard deviation (*s*), and Fisher test (*F*) [61]. The last two parameters gave information about how the correlation between the experimental and calculated activities is affected by the number of compounds in the study (Equation (7)), and what the probability is for the mathematical model to casually occur (Equation (8)).

(6) $$R^{2}=\frac{\sum_{i=1}^{n}{\left({\hat{y}}_{i}-y_{i}\right)}^{2}}{\sum_{i=1}^{n}{\left(y_{i}-\overset{-}{y}\right)}^{2}}$$

(7) $$s=\frac{\sum_{i=1}^{n}{\left({\hat{y}}_{i}-y_{i}\right)}^{2}}{n-2}$$

(8) $$F=\frac{\sum_{i=1}^{n}{\left({\hat{y}}_{i}-\overset{-}{y}\right)}^{2}/{df}_{M}}{\sum_{i=1}^{n}{\left({\hat{y}}_{i}-y_{i}\right)}^{2}/{df}_{E}}$$

where ${\hat{y}}_{i}$, ȳ, and $y_{i}$ refer to the calculated, average, and experimental values of activities, respectively. Also, ${df}_{M}$ and ${df}_{E}$ (Equation (9)) refer to the degrees of freedom of the model and error, respectively.

(9) $${df}_{E}=n-p-1$$

In the mathematical model, *R*^2^ must possess high values (*R*^2^ ≥ 80), while *s* and *F* should have the smallest and largest possible values, respectively, to ensure that the QSAR model is reliable.

Also, to undertake a more sophisticated validation, we used redundancy (*R^P^*) and overfitting (*R^N^*) rules [62]. From the Pearson correlation matrix, we corroborated that the molecular descriptors are not linearly correlated (Table S1).

The goal of the redundancy rule is to detect models with an excess of good molecular descriptors (*R^P^*) and establish that, if *R^P^ < t^P^*, the model is rejected. Depending on the data, *t^P^* values range from 0.01 to 0.1. *R^P^* is defined by Equation (10).

(10) $$R^{P}=\prod_{j=1}^{P^{+}}\left(1-M_{j}\left(\frac{p}{p-1}\right)\right);\;M_{j}>0\;\mathrm{and}\;0\leq \;R^{P}\leq 1$$

On the other hand, the purpose of the overfitting rule is to detect models with an excess of bad molecular descriptors. This rule stipulates that, if *R^N^* < *t^N^* (*ε*), the model is rejected. The *t^N^* (*ε*) values are calculated by the Equation (11).

(11) $$t^{N}\left(\varepsilon \right)=\frac{p\cdot \varepsilon -R}{p\cdot R}$$

where values range from 0.01 to 0.1 and *p* is the number of variables in the model. *R^N^* is defined by Equation (12).

(12) $$R^{N}=\sum_{j=1}^{P^{-}}M_{j;\;}\;M_{j}<0\;\mathrm{and}\;-1\leq R^{N}\leq 0\;$$

where *M_j_* is defined by Equation (13).

(13) $$M_{j}=\frac{R_{jY}}{R}-\frac{1}{P};\;-\frac{1}{P}\leq M_{j}\leq \frac{p-1}{p}$$

*R_jY_* is the absolute value of the regression coefficient between the *j*th descriptors and the response *Y*.

Moreover, we evaluated the predictive ability of our model by the Leave-One-Out $\left(Q_{LOO}^{2}\right)$ method, in which one compound is removed from the data set and the activity (Y_exp_) is correlated using the rest of the data set. The Equation (14) was employed to calculate $Q_{LOO}^{2}$.

(14) $$Q_{LOO}^{2}=1-\frac{\sum_{i=1}^{n}{\left(y_{i}-{\hat{y}}_{\frac{i}{i}}\right)}^{2}}{\sum_{i=1}^{n}{\left(y_{i}-\overset{-}{y}\right)}^{2}}$$

where ŷ_i/i_ is the predicted value of the activity (Y_pred_).

Applicability domain evaluation was carried out by means of William plot construction, which depends on the leverage values and the standardized error in the calculation. The leverage values (*h*) are obtained from the leverage matrix *H*, which contains information about the descriptors on which the model is built. The leverage matrix *H* is defined as Equation (15).

*H* = *X* · (*X*^*T*^ · *X*)^−1^ · *X*^*T*^ (15)

where *X* is the selected descriptor matrix; *X^T^* is the transpose matrix of *X*; and (*X^T^* · *X*)^−1^ is the inverse of matrix (*X^T^ · X*). The leverage values are the diagonal elements of the H matrix. The warning leverage (h*) is calculated as *h* = 3 *p/n*, where *n* is the number of molecules and *p* is the number of descriptors in the model plus one. If one of the compounds has a leverage value higher than the h*, it will be considered an outlier, this is, out of the applicability domain of the model.

## 4. Conclusions

In this study, Geniposide and its aglycone Genipin were tested against different cervical cancer cell lines (CaLo, CaSki, and INBL) from different FIGO stagings and with the most high-risk HPV types. Geniposide did not exert cytotoxic activity, and Genipin was more active against CaLo (IIB, HPV18) and CaSki (metastatic, HPV16) than against INBL (IVB, HPV18).

Derived from the structure–activity relationship and the molecular similarity analysis, we found that the dipole moment is relevant to the cytotoxic activity against the HeLa cell line. Moreover, we observed that the presence of different functional groups, such as an aldehyde or a carboxylic acid, the hydroxyls in C1 and C8, and the lack of the double bond in C7–C8 influenced the biological activity of the iridoids.

Derived from the QSAR study, we obtained a model with a good prediction of the biological activity against HeLa, that reveals the dipole moment as a principal descriptor that impacts on the activity of the iridoids. The designed iridoids incorporated the most relevant structural characteristics of simple iridoids based on the Genipin structure. Therefore, we propose ten of the designed iridoids (D9, D10, D35, D36, D55, D56, D58, D60, D61, and D62), since their predicted activity was lower (0.002–0.146 µM) than the activity of the most cytotoxic iridoid reported in the literature (0.2 µM), according to our QSAR model.

## Figures and Tables

**Table 1:** Cytotoxic activity of Geniposide and Genipin in cervical cancer cell lines (IC_50_: µM).

Cell Lines	Geniposide	Genipin	Cisplatin	Podophyllotoxin
CaSki	>1000	65.930 ± 4.420	0.640 ± 0.080	0.025 ± 0.001
CaLo	>1000	58.970 ± 9.040	0.240 ± 0.020	0.014 ± 0.002
INBL	>1000	178.800 ± 12.990	1.340 ± 0.160	0.011 ± 0.001
HaCaT ^1^	>1000	106.250 ± 9.290	1.150 ± 0.140	0.050 ± 0.006

^1^ HaCaT is a non-tumorigenic cell line of immortalized keratinocytes and was used as control.

**Table 2:** Cytotoxic iridoids against cervical cancer HeLa cells.

Compound	Cell Line	Exposure Time (h)	IC_50_ (µM)
Euphrasin ^1^	HeLa	48	0.20
Campsinol ^1^	HeLa	48	1.90
Artselaenin B ^1^	HeLa	48	12.30
Artselaenin A ^1^	HeLa	48	13.80
Pulchelloside I ^2^	HeLa	48	25.22
Catalpol ^3^	HeLa	48	28.20
Lamiide ^2^	HeLa	48	31.96
Spinomannoside ^2^	HeLa	48	38.89
5-deoxypulchelloside I ^2^	HeLa	48	42.47
Geniposidic acid ^4^	HeLa	48	43.60
10-*O*-(E)-*p*-coumaroylgeniposidic acid ^3^	HeLa	48	48.10
Genipin	HeLa	48	419.00 ^5^
Geniposide	HeLa	48	NA ^5^

^1^ Compounds from [31]; ^2^ compounds from [39]; ^3^ compounds from [32]; ^4^ compounds from [33]; ^5^ experimental data. NA: not active.

**Table 3:** Molecular descriptors of simple iridoids with reported cytotoxic activity (IC_50_) against the HeLa cell line.

Ligand	IC_50_ (µM)	GAP_HOMO-LUMO_ (kcal)	Dipole moment (debye)	Area (Å^2^)	Volume (Å^3^)	PSA (Å^2^)	Ovality	LogP	Polarizability	HBD Count	HBA Count
Euphrasin	0.20	−123.01	4.81	234.27	216.65	44.645	1.34	−0.10	57.69	1	4
Campsinol	1.90	−122.30	5.78	236.86	216.59	46.922	1.36	−0.10	57.69	1	4
Artselaenin B	12.3	−125.40	4.67	227.13	208.64	26.808	1.34	1.11	57.01	0	3
Artselaenin A	13.8	−121.90	4.20	228.14	209.46	27.236	1.34	1.11	57.11	0	3
Genipin	419.00	−126.18	1.26	239.61	219.74	64.589	1.36	−0.26	57.91	2	5

**Table 4:** Molecular descriptors of simple iridoids with reported cytotoxic activity against the HeLa cell line.

Ligand	IC_50_ (µM)	GAP_HOMO-LUMO_ (kcal)	Dipole Moment (debye)	Area (Å^2^)	Volume (Å^3^)	PSA (Å^2^)	Ovality	LogP	Polarizability	HBD Count	HBA Count
Genipin	419.00	−126.18	1.26	239.61	219.74	64.589	1.36	−0.26	57.91	2	5
Geniposide	NA	−128.37	3.41	371.47	355.38	119.843	1.53	−2.00	68.89	5	9

**Table 5:** Calculated molecular descriptor values and experimental biological activity (IC_50_) of iridoid glycosides and Geniposide tested against the HeLa cell line.

Ligand	IC_50_ (µM)	GAP_HOMO-LUMO_ (kcal)	Dipole Moment (debye)	Area (Å^2^)	Volume (Å^3^)	PSA (Å^2^)	Ovality	LogP	Polarizability	HBD Count	HBA Count
Pulchelloside I	25.22	−121.84	5.95	383.69	372.30	148.544	1.53	−3.30	70.33	7	11
Catalpol	28.20	−144.80	2.50	324.51	320.55	126.832	1.43	−3.24	65.89	6	10
Laamide	31.96	−116.96	7.99	392.42	373.46	158.057	1.56	−3.48	70.47	7	11
Spinomannoside	38.89	−124.51	4.28	381.33	367.08	140.269	1.54	−2.51	69.87	5	10
5-deoxypulchelloside I	42.47	−125.49	3.32	374.98	365.07	134.805	1.52	−2.51	69.70	6	10
Geniposidic acid *	43.60	−85.84	12.02	353.50	335.17	139.852	1.51	--	67.68	5	10
10-*O*-(E)-*p*-coumaroylgeniposidic acid *	48.10	−61.97	16.64	481.01	476.36	147.201	1.63	--	79.38	5	11
Geniposide	NA	−128.37	3.41	371.47	355.38	119.843	1.53	−2.00	68.89	5	9

* These iridoids were analyzed in their anion form.

**Table 6:** Magnitude and polar spherical coordinates of the dipole vector of simple active iridoids and their experimental biological activity values (IC_50_).

Ligand	ρ (debye)	θ	φ	IC_50_ (µM)
Euphrasin	4.81	0.8097	1.6749	0.20
Campsinol	5.78	−1.0080	1.3527	1.90
Artselaenin B	4.67	0.9716	1.3933	12.3
Artselaenin A	4.20	−1.3515	1.1341	13.8
Genipin	1.26	1.5078	2.2265	419.00

**Table 7:** Values of the molecular descriptors present in the QSAR model and the biological activity (IC_50_) of simple and glycosylated iridoids.

Ligand	ρ (debye)	$∆PSA({Å}^{2})$	LogS	${∆Polarizability}^{2}$	${IC}_{50}(µM)$
Euphrasin	4.81	−19.944	−1.21	0.048	0.20
Campsinol	5.78	−17.667	−1.21	0.048	1.90
Artselaenin B	4.67	−37.781	−2.23	0.810	12.30
Artselaenin A	4.20	−37.353	−2.23	0.640	13.80
Pulchelloside I	5.95	83.955	−0.50	154.256	25.22
Catalpol	2.50	62.243	−0.19	63.680	28.20
Lamiide	7.99	93.468	−0.69	157.754	31.96
Spinomannoside	4.28	75.680	−0.73	143.042	38.89
5-deoxypulchelloside I	3.32	70.216	−0.73	139.004	42.47
Geniposidic acid ^1^	12.02	75.260	−1.22	95.453	43.60
10-*O*-(E)-*p*-coumaroylgeniposidic acid ^1^	16.64	82.610	−2.86	460.961	48.10
Genipin	1.26	0	−1.50	0	419.00

^1^ These iridoids were analyzed in their anion form.

**Table 8:** Values of the cytotoxic experimental Y_exp_, calculated Y_calc_, and predicted Y_pred_ activities, and residual_calc_ and residual_pred_ values are shown.

Ligand	Y_exp_	Y_calc_	Y_pred_	Hat	residual_calc_	residual_pred_
Euphrasin	0.70	0.19	−0.24	0.451	−0.51	−0.93
Campsinol	−0.28	0.12	0.40	0.403	0.40	0.68
Artselaenin B	−1.09	−1.09	−1.10	0.389	0	−0.01
Artselaenin A	−1.14	−1.18	−1.21	0.377	−0.04	−0.07
Pulchelloside I	−1.40	−1.09	−0.95	0.305	0.32	0.46
Catalpol	−1.45	−1.71	−2.00	0.524	−0.26	−0.55
Lamiide	−1.50	−1.95	−2.25	0.407	−0.44	−0.75
Spinomannoside	−1.59	−1.57	−1.57	0.231	0.02	0.02
5-deoxypulchelloside I	−1.63	−1.39	−1.32	0.244	0.24	0.31
Geniposidic acid ^1^	−1.64	−1.59	−1.56	0.387	0.05	0.08
10-*O*-(E)-*p*-coumaroylgeniposidic acid ^1^	−1.68	−1.62	−1.51	0.656	0.06	0.17
Genipin	−2.62	−2.44	−2.13	0.627	0.18	0.49

^1^ These iridoids were analyzed in their anion form.

**Table 9:** Molecular descriptors of the best designed iridoids.

Ligand	GAP_HOMO-LUMO_ (kcal)	Dipole Moment (debye)	Area (Å^2^)	Volume (Å^3^)	PSA (Å^2^)	Ovality	LogP	Polarizability	HBD Count	HBA Count
D9	−123.61	4.80	187.58	170.55	42.052	1.26	0.42	53.94	1	3
D10 ^1^	−599.13	12.32	190.51	175.42	54.222	1.26	--	54.14	1	4
D35	−123.99	3.36	197.34	178.62	60.872	1.29	−0.67	54.59	2	4
D36	−138.78	0.69	196.25	176.32	47.478	1.29	0.55	54.25	2	3
D55	−139.52	1.70	196.35	176.4	47.351	1.29	0.61	54.25	2	3
D56	−118.90	4.81	213.74	196.37	57.202	1.31	−0.41	56.08	2	4
D58	−140.01	1.99	219.39	201.1	61.709	1.32	−0.28	56.25	3	4
D60	−144.58	2.30	201.54	184.95	73.770	1.28	−1.50	54.89	3	5
D61	−145.63	3.19	207.72	190.41	80.817	1.30	−1.25	55.33	4	5
D62	−135.25	0.86	196.11	178.97	61.665	1.28	−0.79	54.50	3	4

^1^ These iridoids were analyzed in their anion form.

## Data Availability

Data are contained within the article and Supplementary Materials.

## Supplementary Materials

The following supporting information can be downloaded at: https://www.mdpi.com/article/10.3390/ph16121647/s1, Figure S1: Structure of iridoid skeleton. Principal substituents in R_2_ and R_6_ are specified.; Figure S2: The 2D structures of iridoids with reported cytotoxic activity against the HeLa cell line.; Figure S3: The 3D structures of iridoid glycosides evaluated against the HeLa cell line, including the vector dipole (gold arrow). (A) Geniposidic acid, (B) 10-*O*-(E)-*p*-coumaroylgeniposidic acid, (C) Catalpol, (D) Geniposide, (D) Lamiide, (F) Pulchelloside I, (G) 5-deoxypulchelloside I, (H) Spinomannoside. Figure S4: Molecular electrostatic potential iso-surface of −10 kcal/mol of iridoid glycosides. (A) Pulchelloside I, (B) Catalpol, (C) Lamiide, (D) Spinomannoside, (E) 5-deoxypulchelloside I, (F) Geniposidic acid, (G) 10-*O*-(E)-*p*-coumaroylgeniposidic acid, (H) Geniposide. Figure S5: Molecular electrostatic potential map of iridoid glycosides. (A) Pulchelloside I, (B) Catalpol, (C) Lamiide, (D) Spinomannoside, (E) 5-deoxypulchelloside I, (F) Geniposidic acid, (G) 10-*O*-(E)-*p*-coumaroylgeniposidic acid, (H) Geniposide. Figure S6: Alignment of Genipin with Geniposide. (A) Common similarity centers by CFDs of Genipin (purple circles represent the CFDs selected for the alignment). (B) Dipole vector represented by gold arrows of both iridoids aligned is shown (a: Genipin, b: Geniposide). Figure S7: The 3D structures of the best designed iridoids based on Genipin. Gold arrows represent dipole vector in each iridoid. (A) D9, (B) D10, (C) D35, (D) D36, (E) D55, (F) D56, (G) D58, (H) D60, (I) D61, (J) D62. Figure S8: Molecular electrostatic potential iso-surface of −10 kcal/mol of the best designed iridoids. (A) D9, (B) D10, (C) D35, (D) D36, (E) D55, (F) D56, (G) D58, (H) D60, (I) D61, (J) D62. Figure S9: Molecular electrostatic potential map of the best designed iridoids. (A) D9, (B) D10, (C) D35, (D) D36, (E) D55, (F) D56, (G) D58, (H) D60, (I) D61, (J) D62. Figure S10: Independent correlations between the biological activity and the descriptors of the QSAR model. (A) −Log IC_50_ vs Dipole moment, (B) −Log IC_50_ vs ∆PSA, (C) −Log IC_50_ vs LogS, (D) −Log IC_50_ vs ∆Polarizability^2^. Table S1: Pearson correlation matrix. Table S2: The pKa values of the acid iridoids. Table S3: Molecular descriptors of designed iridoids.
