# Supplementary material for: Ligand-Based Drug Design of Genipin Derivatives with Cytotoxic Activity against HeLa Cell Line: A Structural and Theoretical Study
Source: Pharmaceuticals (Basel). 2023 Nov 23;16(12):1647. doi: 10.3390/ph16121647 (PMC10748106; doi:10.3390/ph16121647)
Supplement: Supplementary file 1 [file pharmaceuticals-16-01647-s001.zip › pharmaceuticals-2680046-supplementary.pdf]

Supplementary data

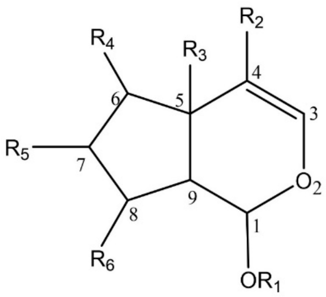

where
R_2: -$COOCH_3$,
-COOH,
-COH.
R_6: -CH_3,
-OH,
-CH_2OH.

Figure S1. Structure of iridoid skeleton. Principal substituents in R2 and R6 are specified.

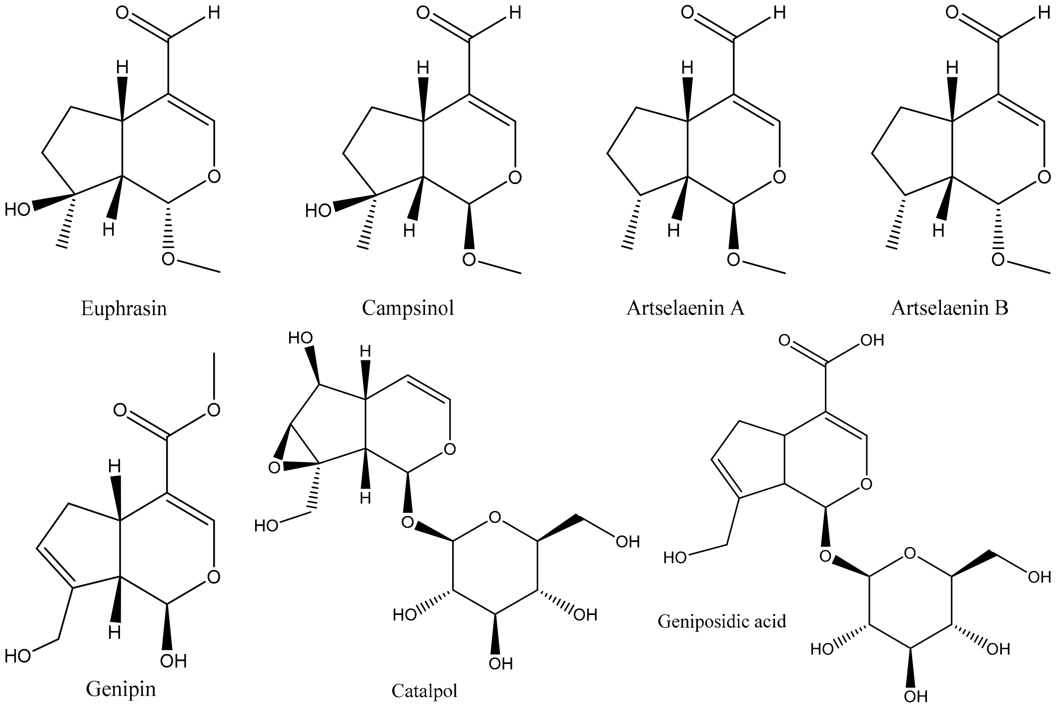

Figure S2. *Cont.*

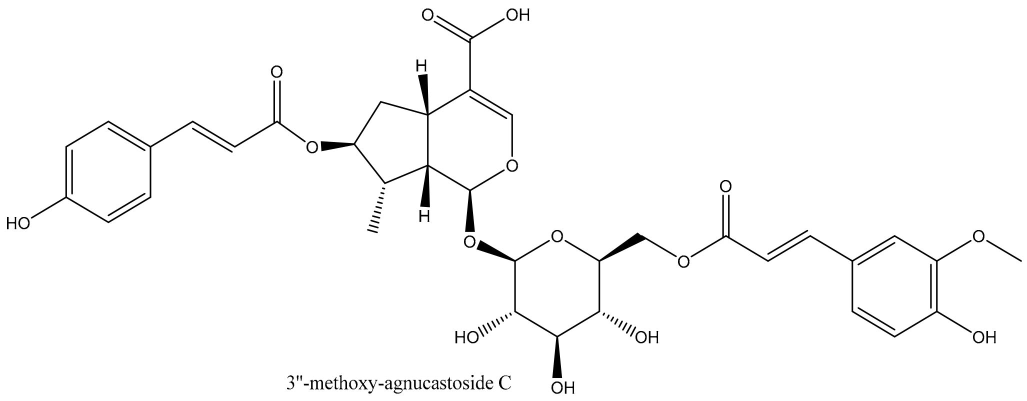

3''-methoxy-agnucastoside C

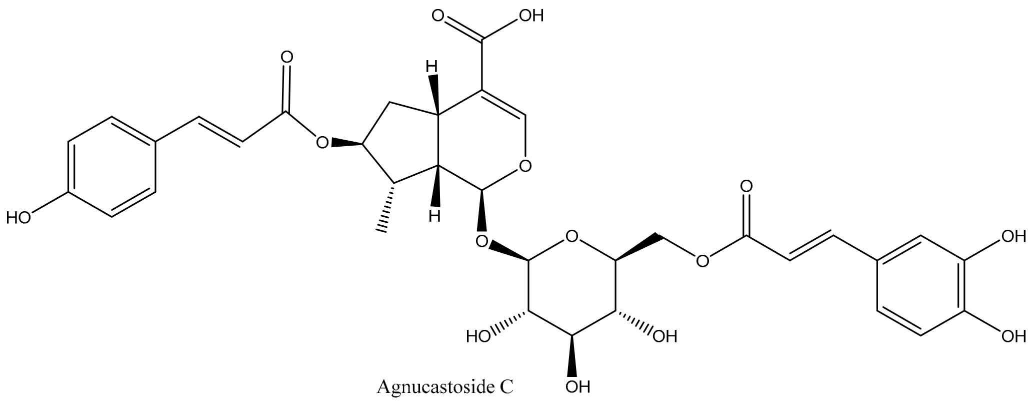

Agnucastoside C

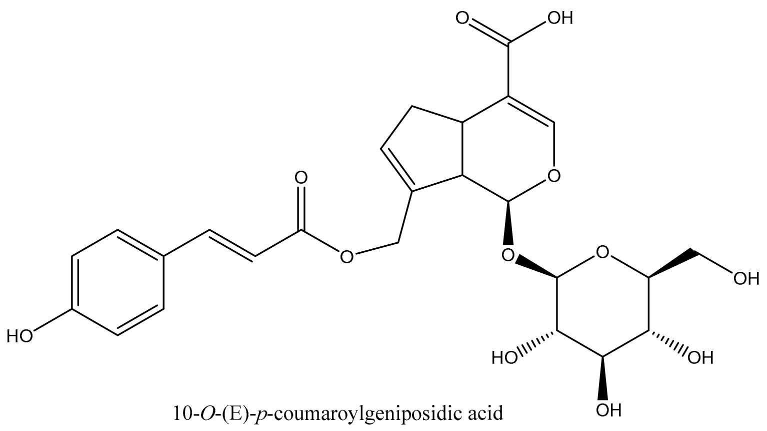

10-*O*-(E)-*p*-coumaroylgeniposidic acid

Figure S2. *Cont.*

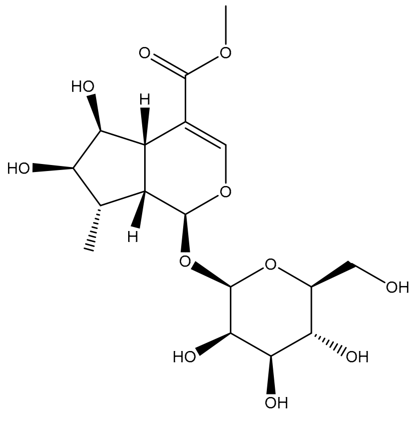

Spinomannoside

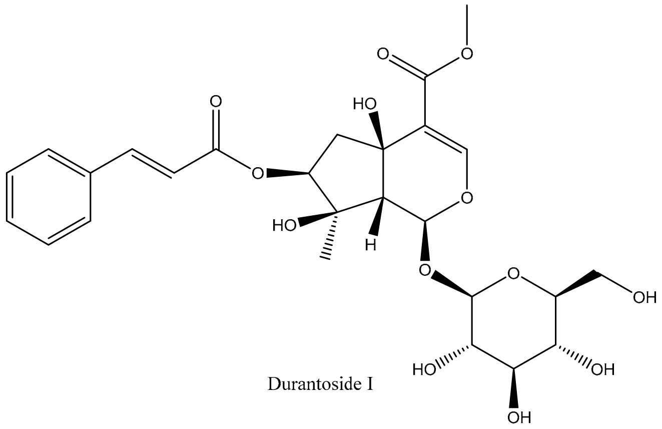

Durantoside I

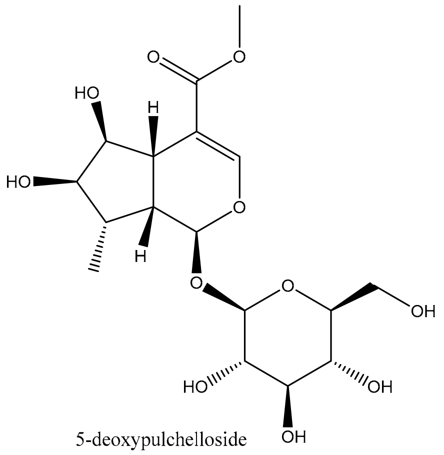

5-deoxypulchelloside

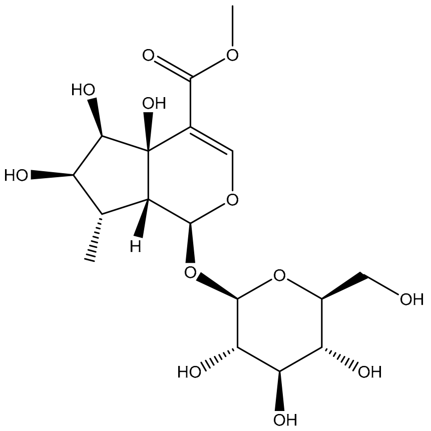

Pulchelloside

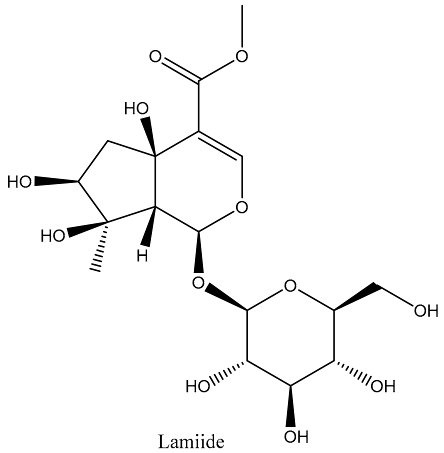

Lamiide

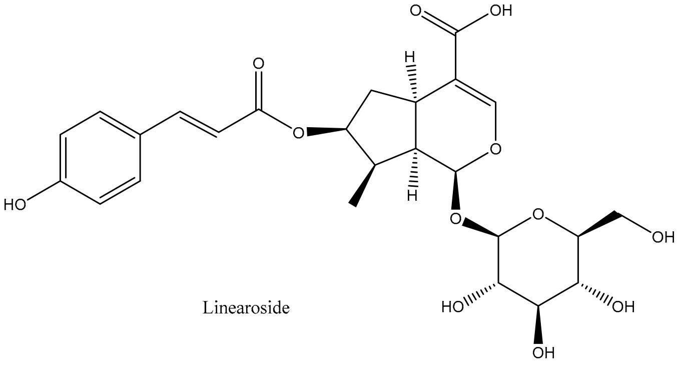

Linearoside

Figure S2. *Cont.*

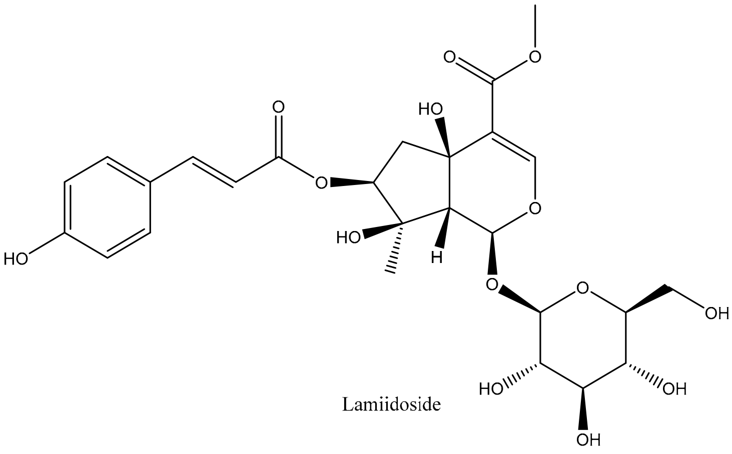

Lamiidoside

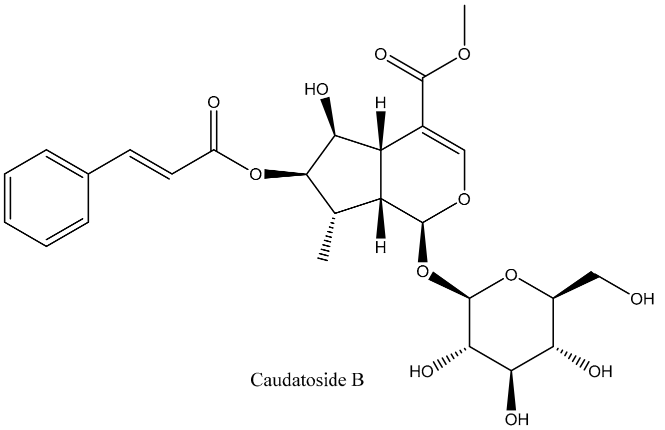

Caudatoside B

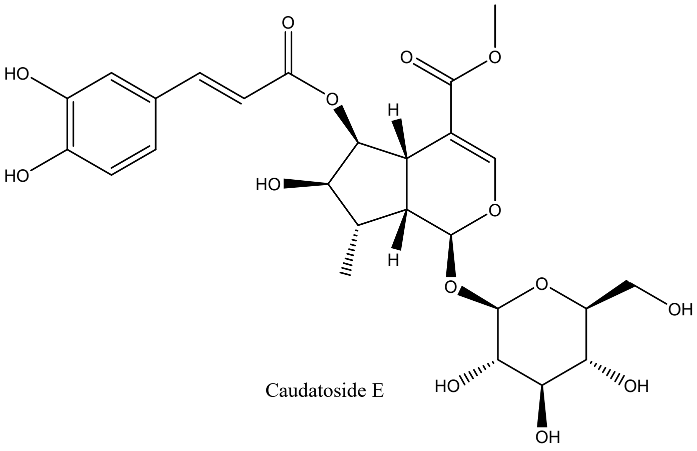

Caudatoside E

Figure S2. *Cont.*

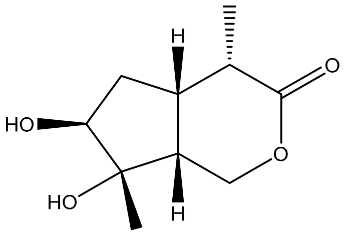

Isopatriscabrol

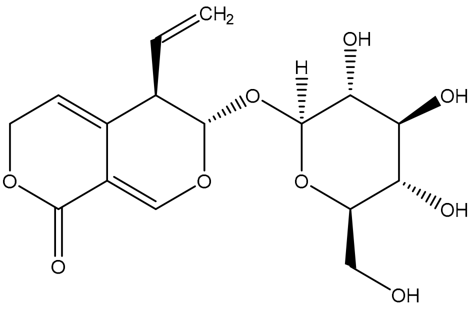

Gentiopicroside

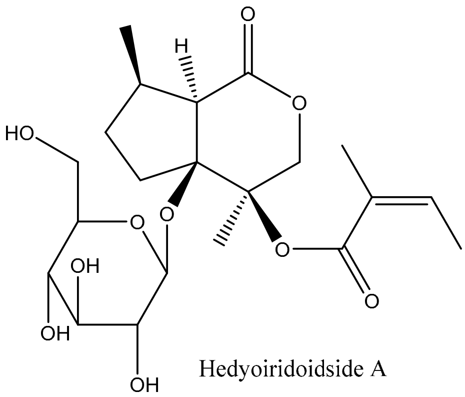

Hedyoiridoidside A

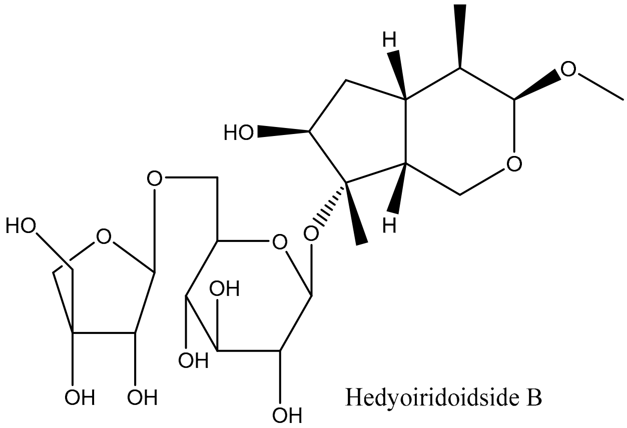

Hedyoiridoidside B

Figure S2. *Cont.*

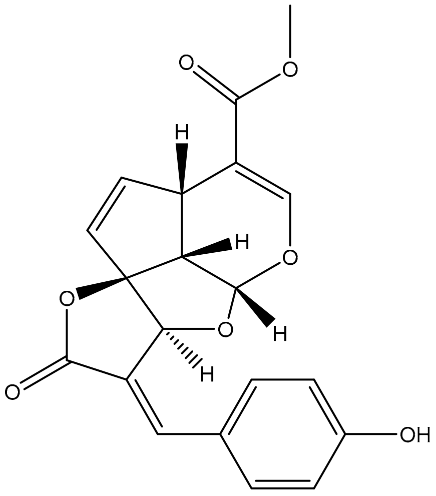

Prismatomerin

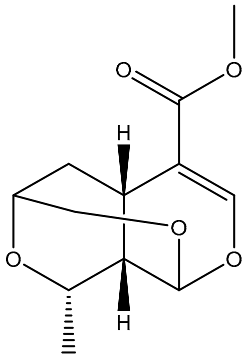

Sarracenin

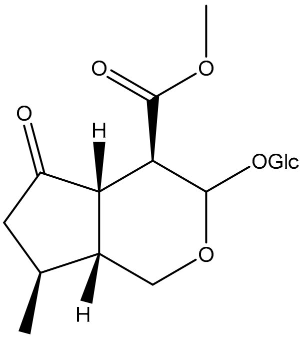

Loniceroside A

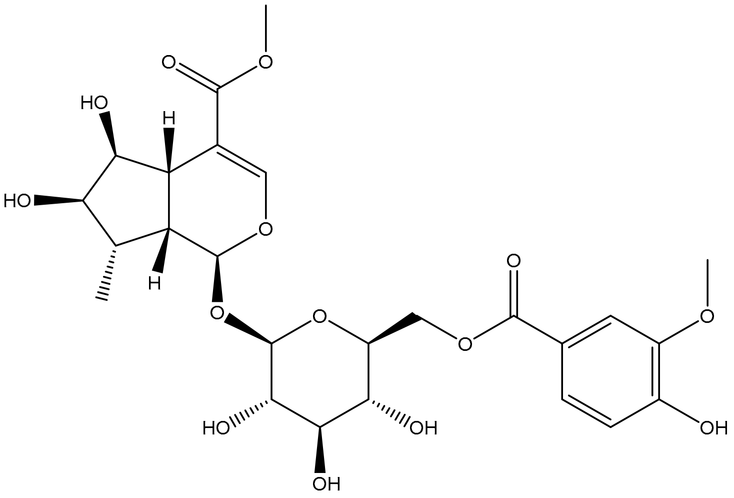

Tunispinoside A

Figure S2. *Cont.*

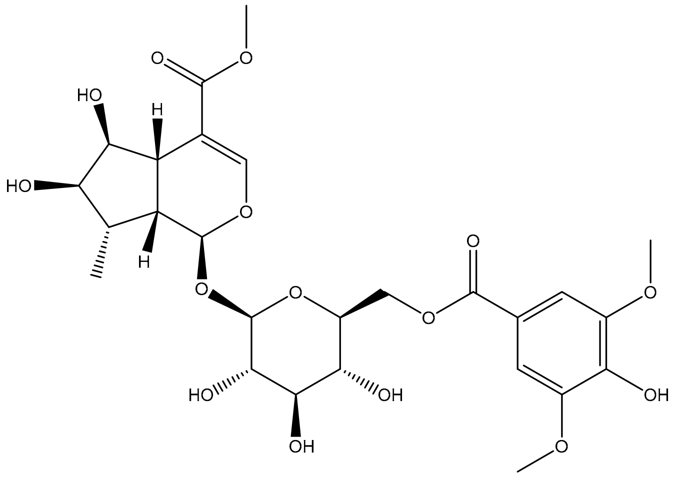

Tunispinoside B

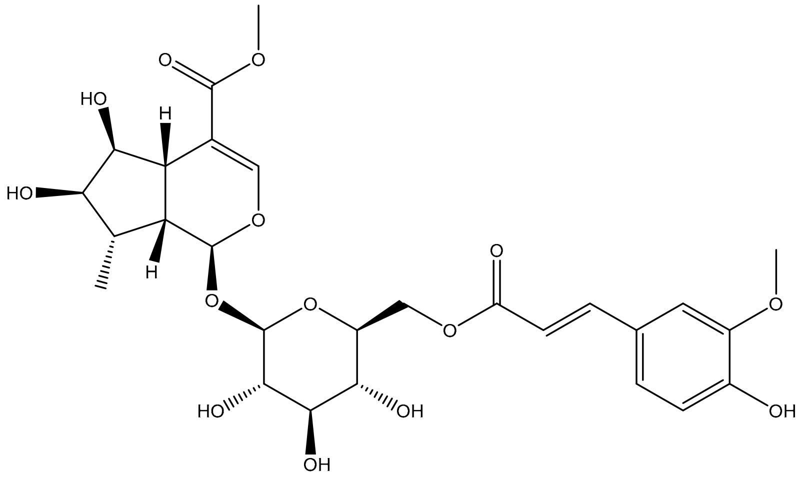

Tunispinoside C

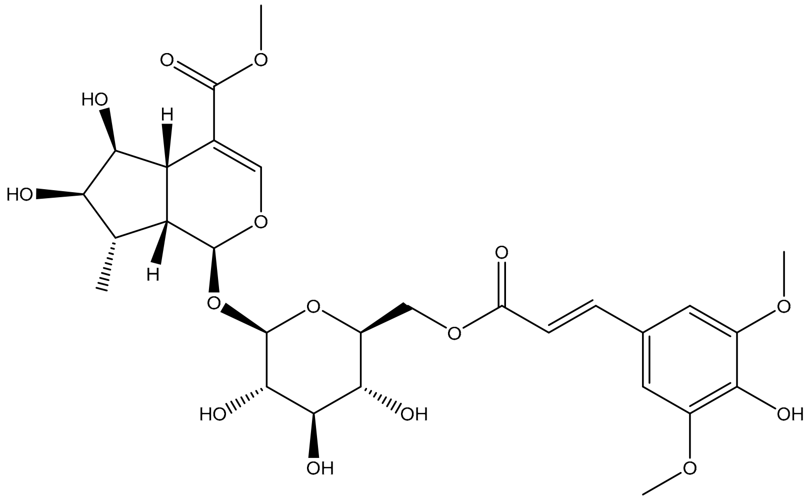

Tunispinoside D

Figure S2. *Cont.*

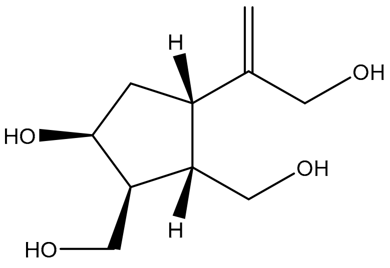

Dioscoridin A

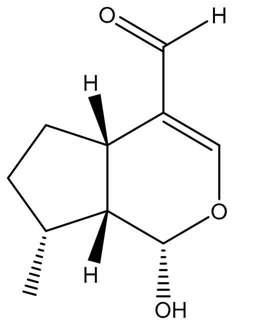

1-epi-bosnarol

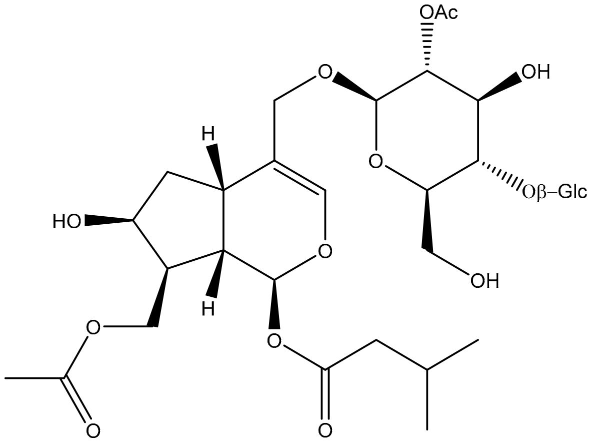

Dioscoridin B

Figure S2. *Cont.*

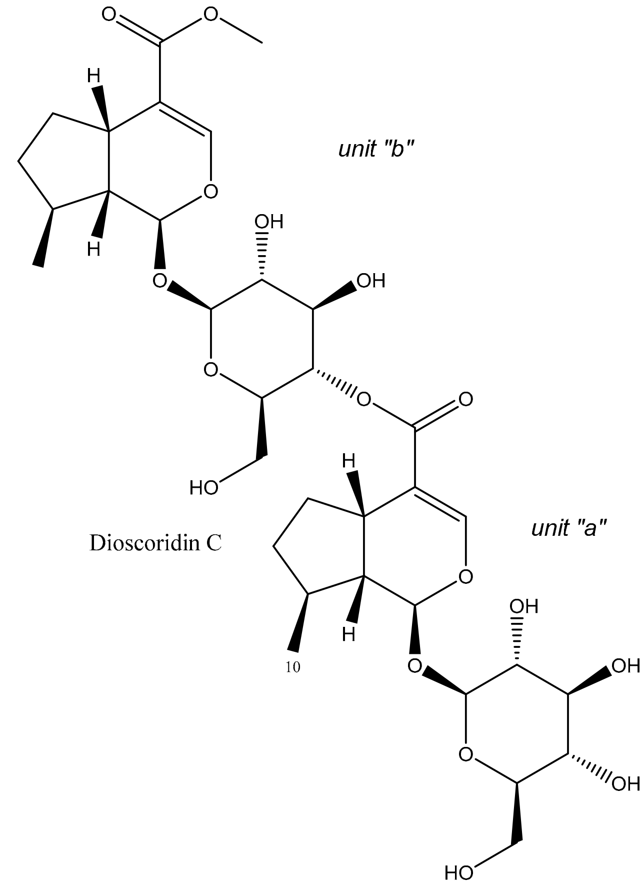

Dioscoridin C

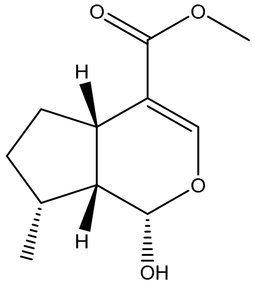

8-epi-deoxyloganin aglycone

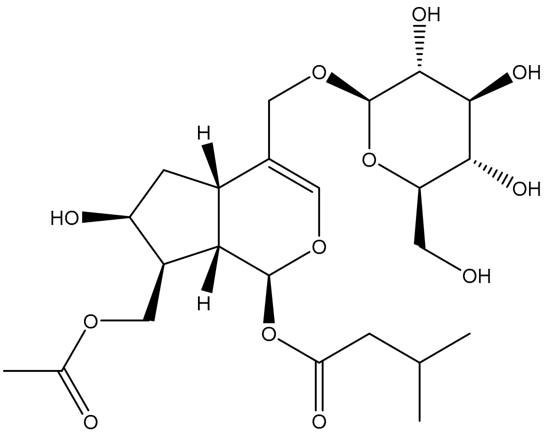

10-acetylpatrionoside

Figure S2. *Cont.*

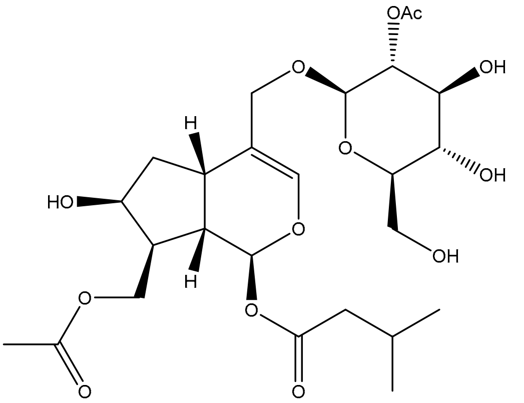

10,2'-diacetylpatrinoside

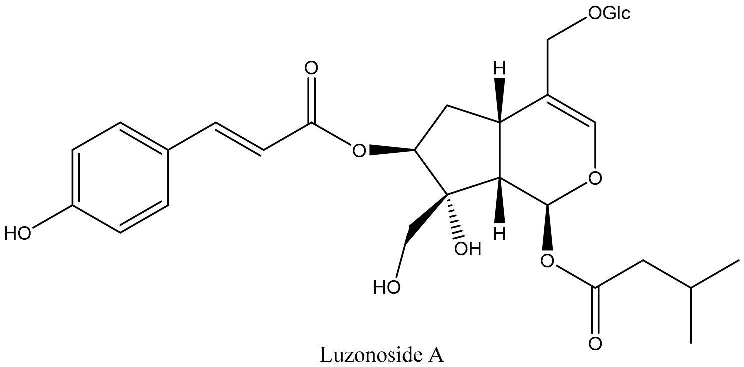

Luzonoside A

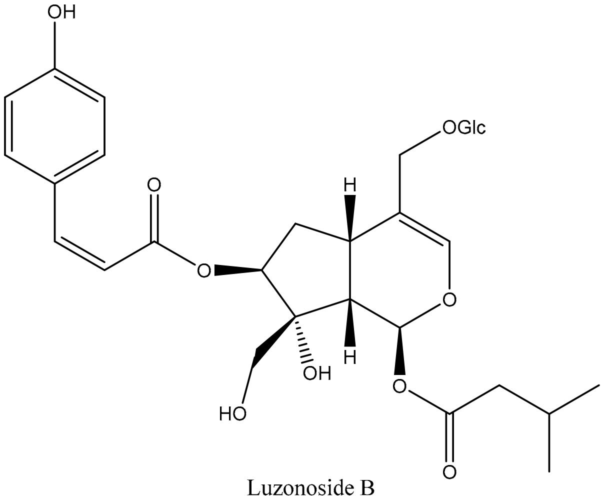

Luzonoside B

Figure S2. *Cont.*

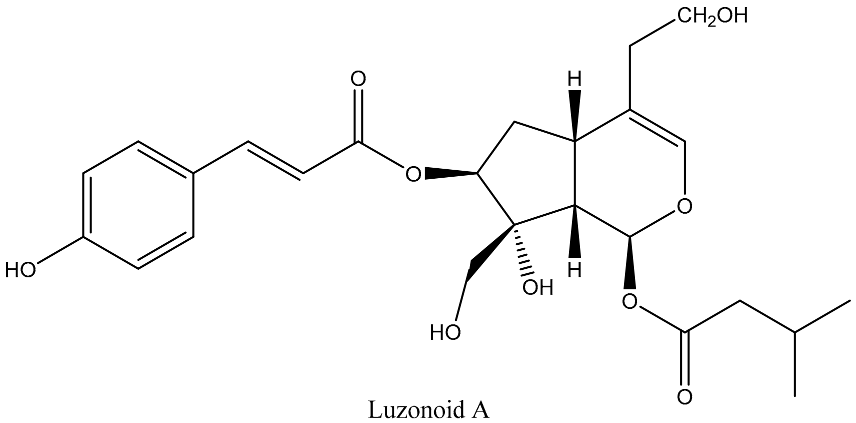

Luzonoid A

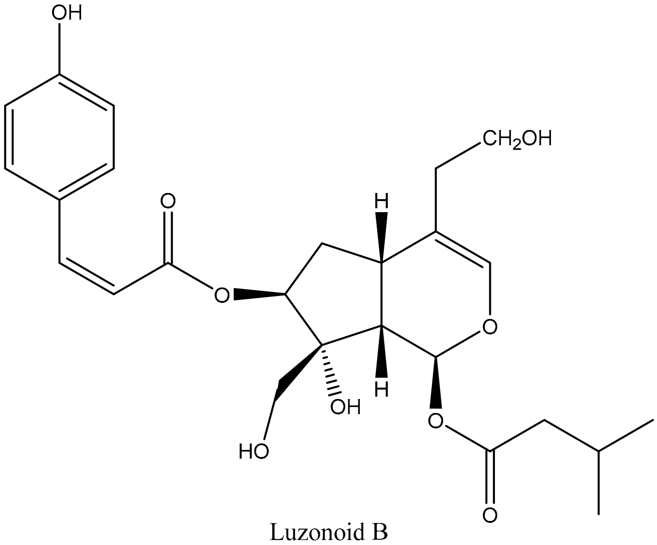

Luzonoid B

Figure S2. *Cont.*

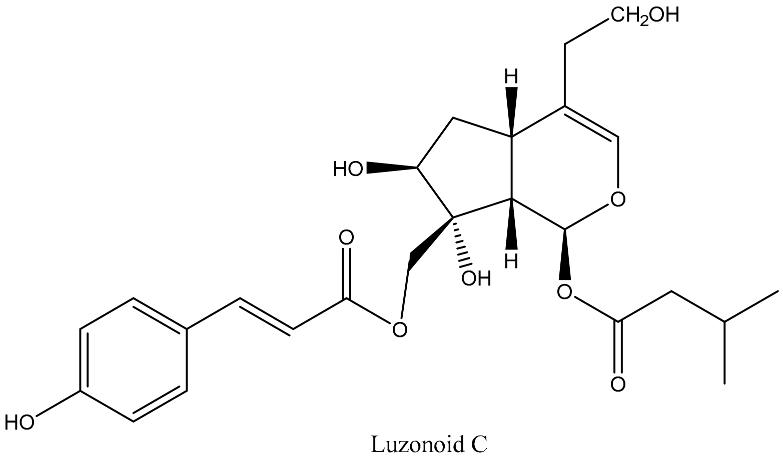

Luzonoid C

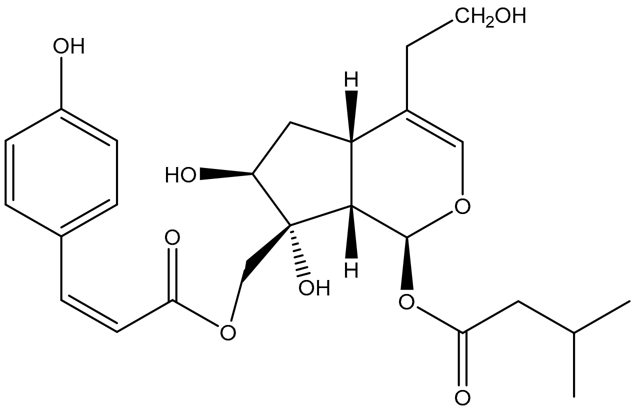

Luzonoid D

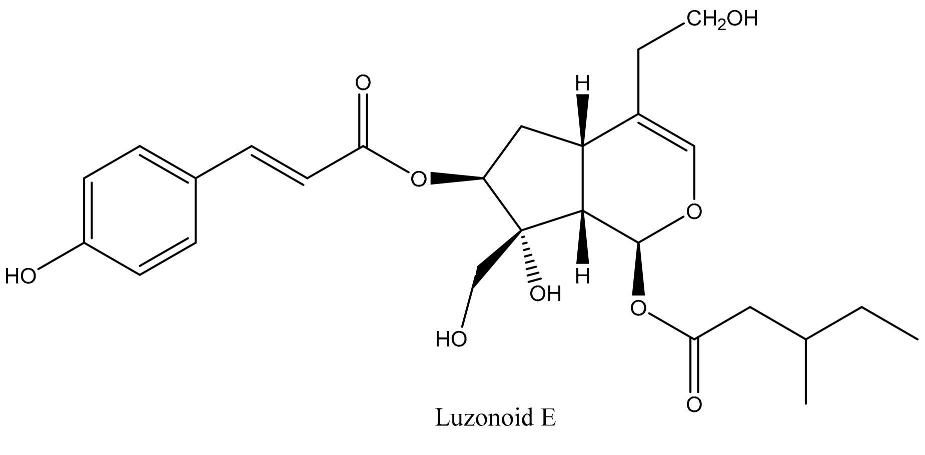

Luzonoid E

Figure S2. 2D structures of iridoids with reported cytotoxic activity against HeLa cell line.

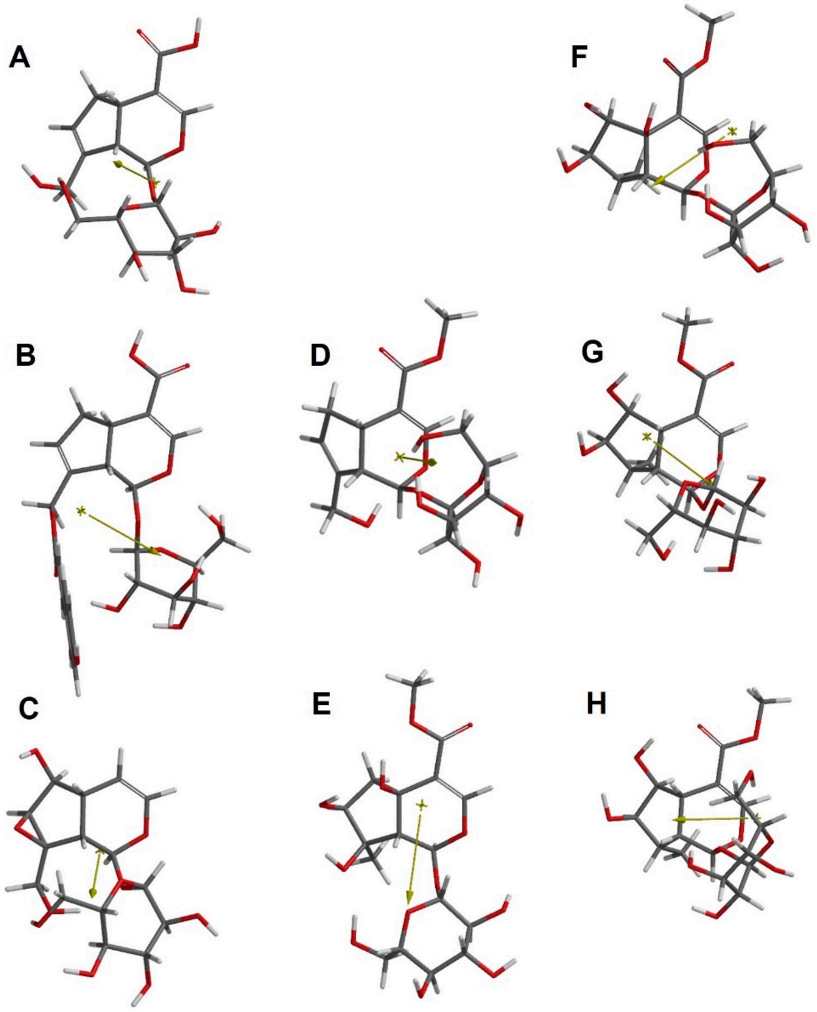

Figure S3. 3D structures of iridoids glycosides evaluated against HeLa cell line, including the dipole vector (gold arrow). (A) Geniposidic acid, (B) 10-*O*-(E)-*p*-coumaroylgeniposidic acid, (C) Catalpol, (D) Geniposide, (E) Lamiide, (F) Pulchelloside I, (G) 5-deoxypulchelloside I, (H) Spinomannoside.

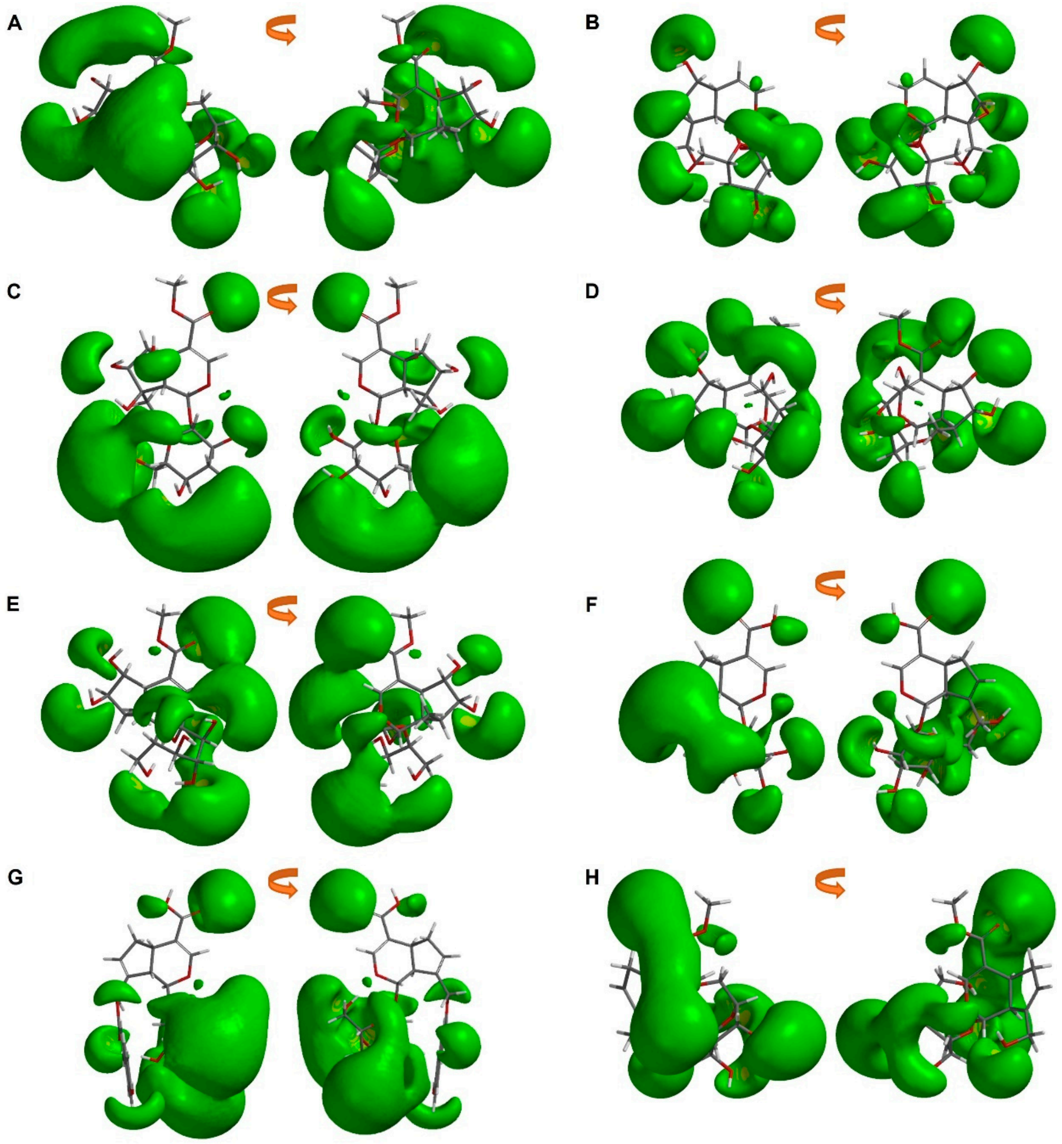

Figure S4. Molecular electrostatic potential iso-surface of -10 kcal/mol of iridoids glycosides. (A) Pulchelloside I, (B) Catalpol, (C) Lamiide, (D) Spinomannoside, (E) 5-deoxypulchelloside I, (F) geniposidic acid, (G) 10-*O*-(E)-*p*-coumaroylgeniposidic acid, (H) Geniposide.

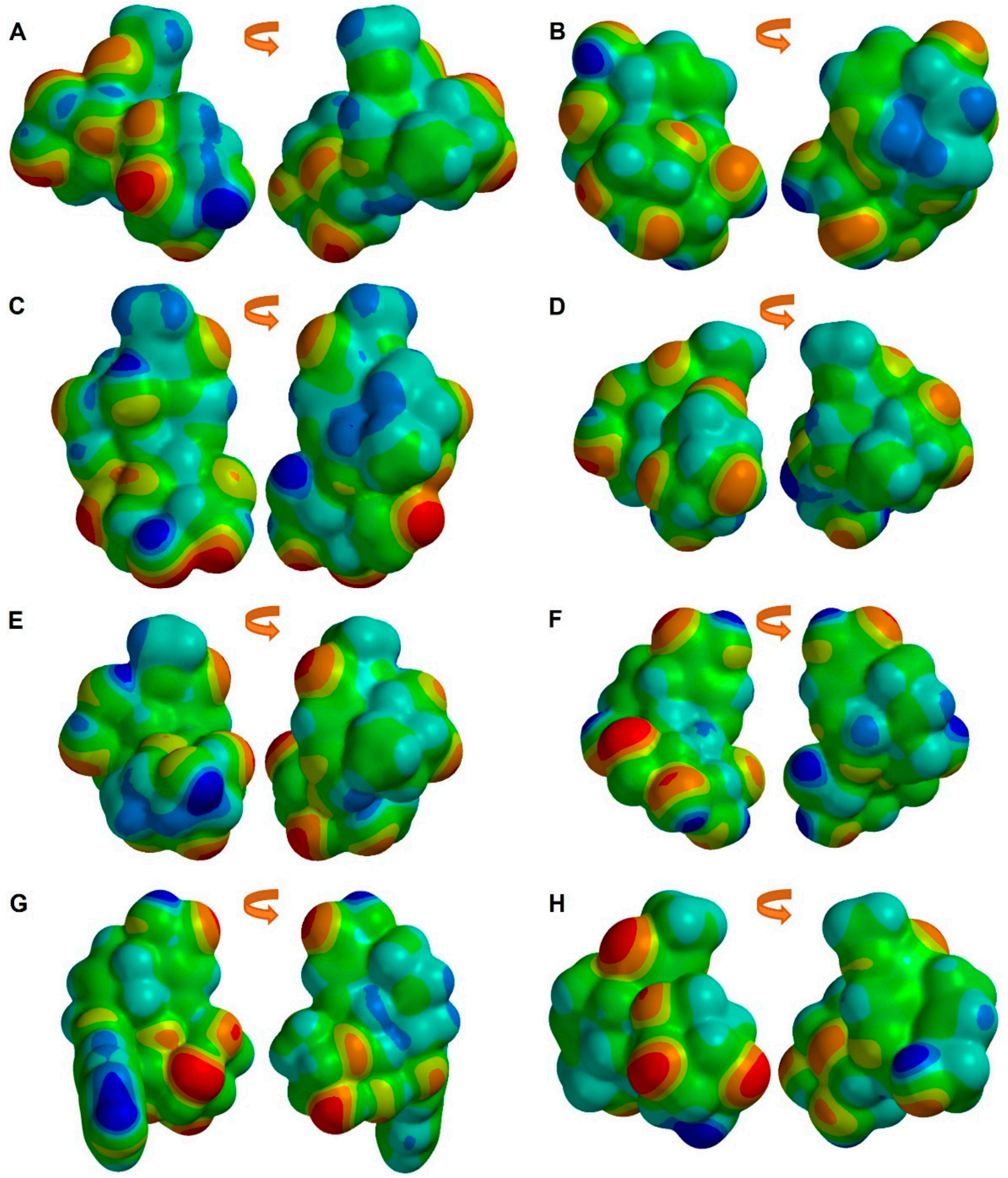

Figure S5. Molecular electrostatic potential map of iridoids glycosides. (A) Pulchelloside I, (B) Catalpol, (C) Lamiide, (D) Spinomannoside, (E) 5-deoxypulchelloside I, (F) Geniposidic acid, (G) 10-*O*-(E)-*p*-coumaroylgeniposidic acid, (H) Geniposide.

A

B

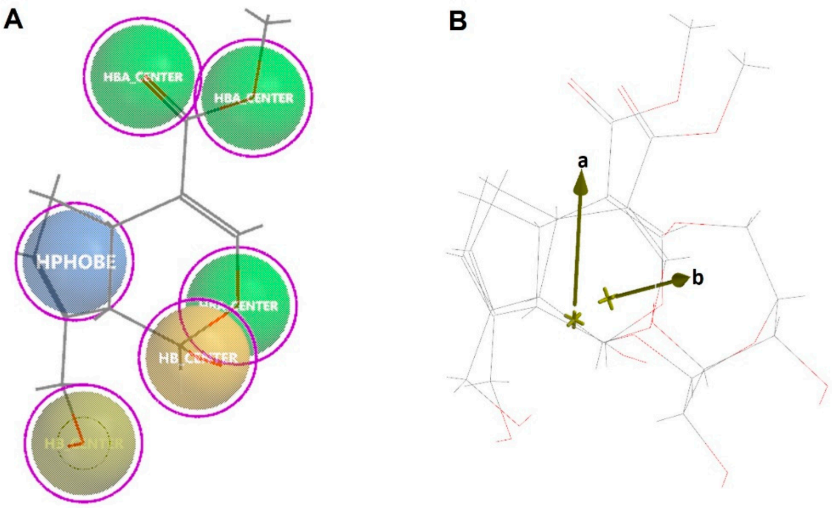

Figure S6. Alignment of genipin with geniposide. (A) Common similarity centers by CFDs of genipin (purple circles represent the CFDs selected for the alignment), (B) Dipole vector represented by gold arrows of both iridoids aligned is shown (a: genipin, b: geniposide).

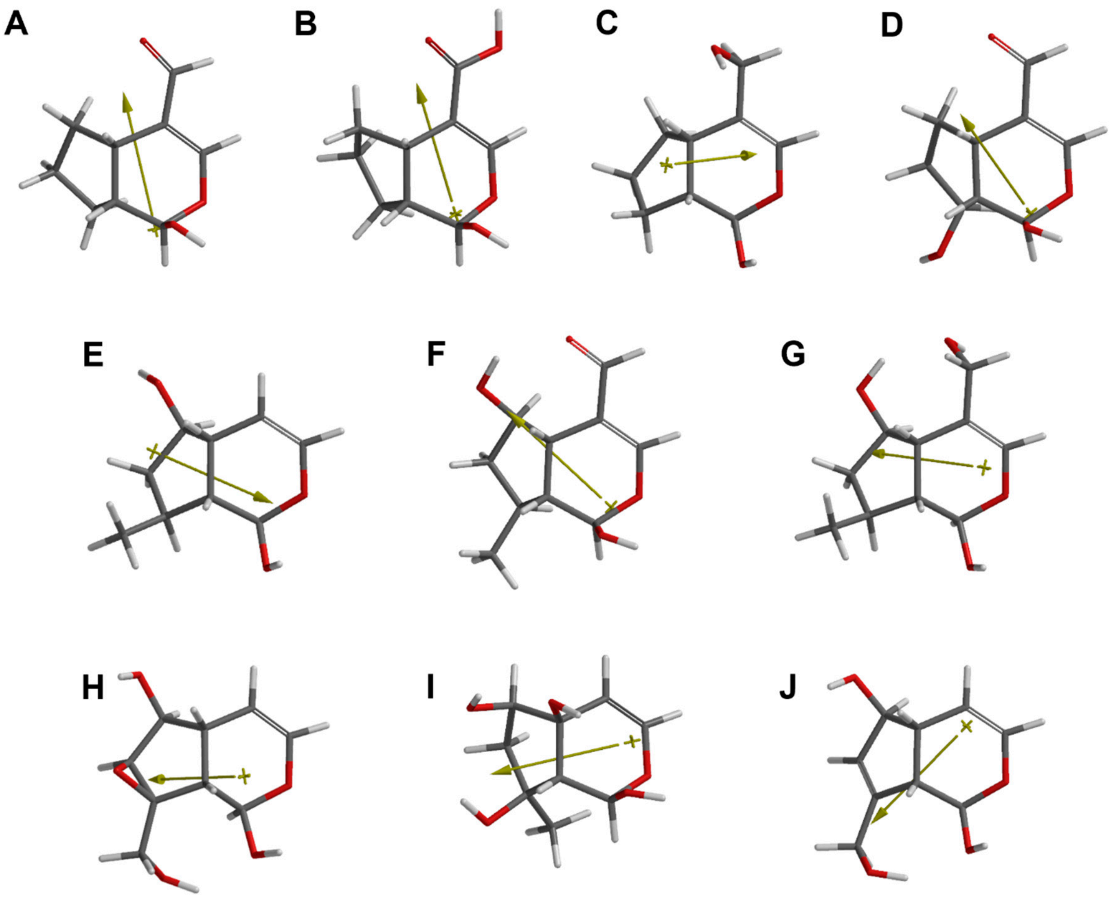

Figure S7. 3D structures of the best designed iridoids based on genipin. Gold arrows represent dipole vector in each iridoid. (A) D9, (B) D10, (C) D35, (D) D36, (E) D55, (F) D56, (G) D58, (H) D60, (I) D61, (J) D62.

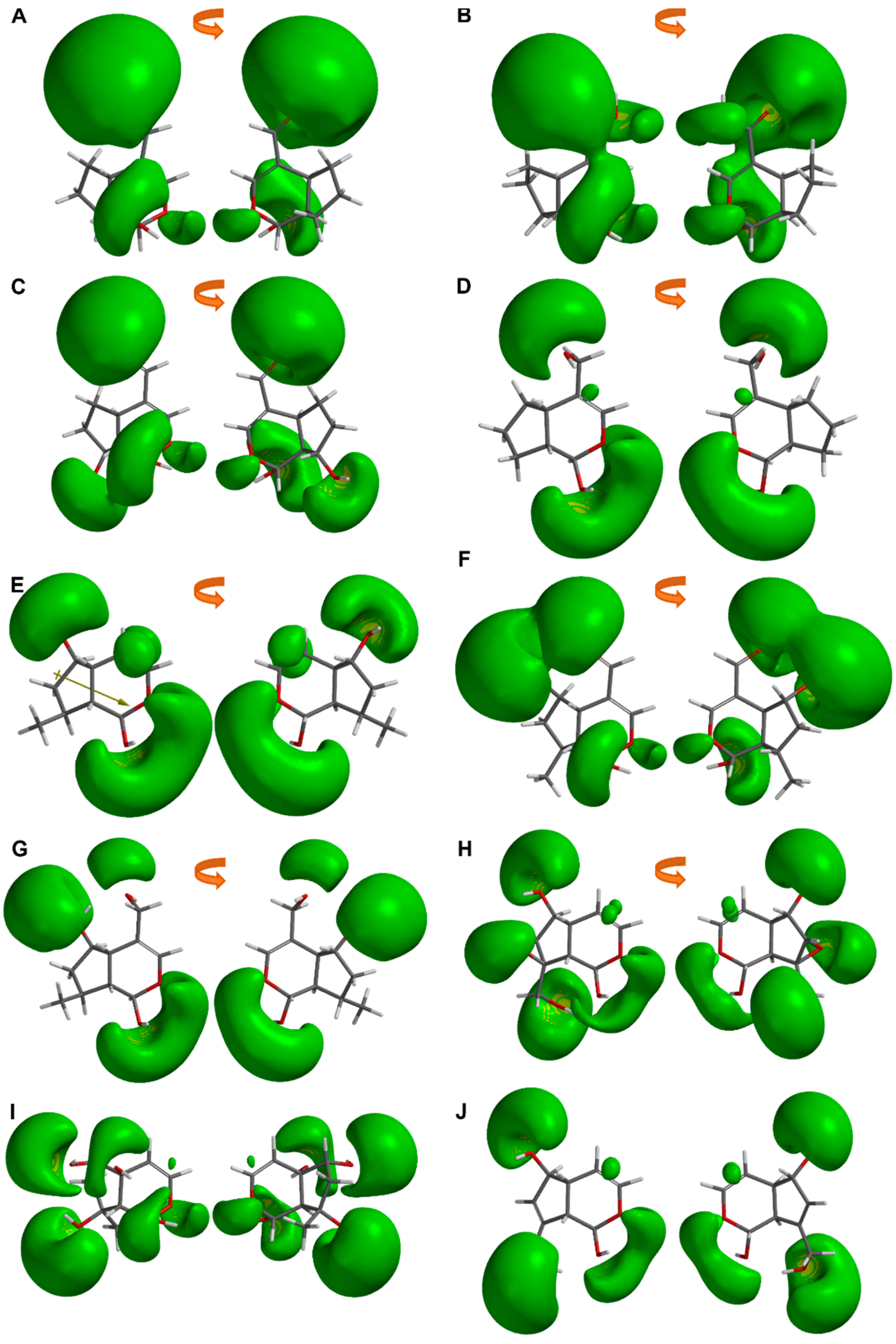

Figure S8. Molecular electrostatic potential iso-surface of -10 kcal/mol of the best designed iridoids. (A) D9, (B) D10, (C) D35, (D) D36, (E) D55, (F) D56, (G) D58, (H) D60, (I) D61, (J) D62.

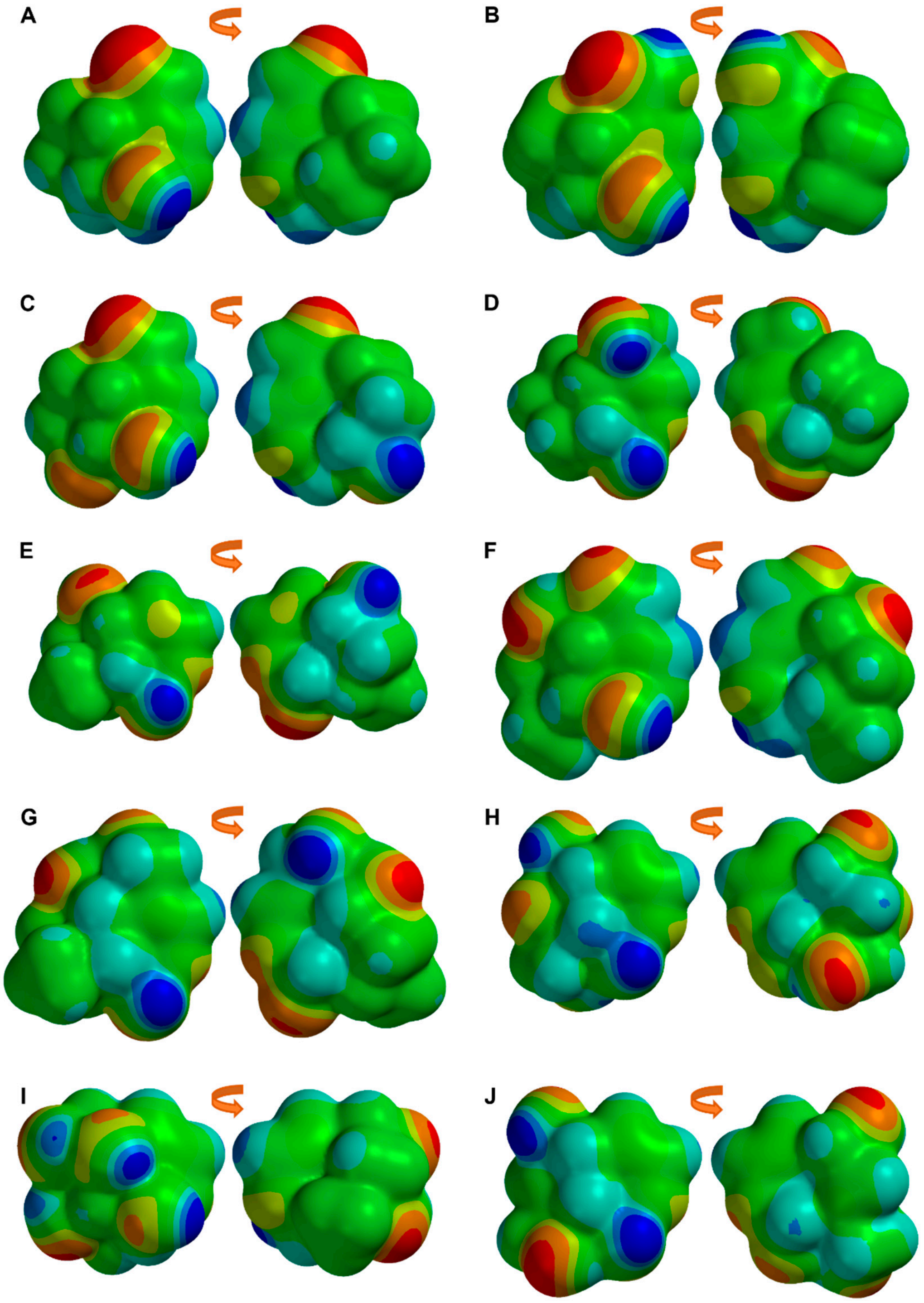

Figure S9. Molecular electrostatic potential map of the best designed iridoids. (A) D9, (B) D10, (C) D35, (D) D36, (E) D55, (F) D56, (G) D58, (H) D60, (I) D61, (J) D62.

.

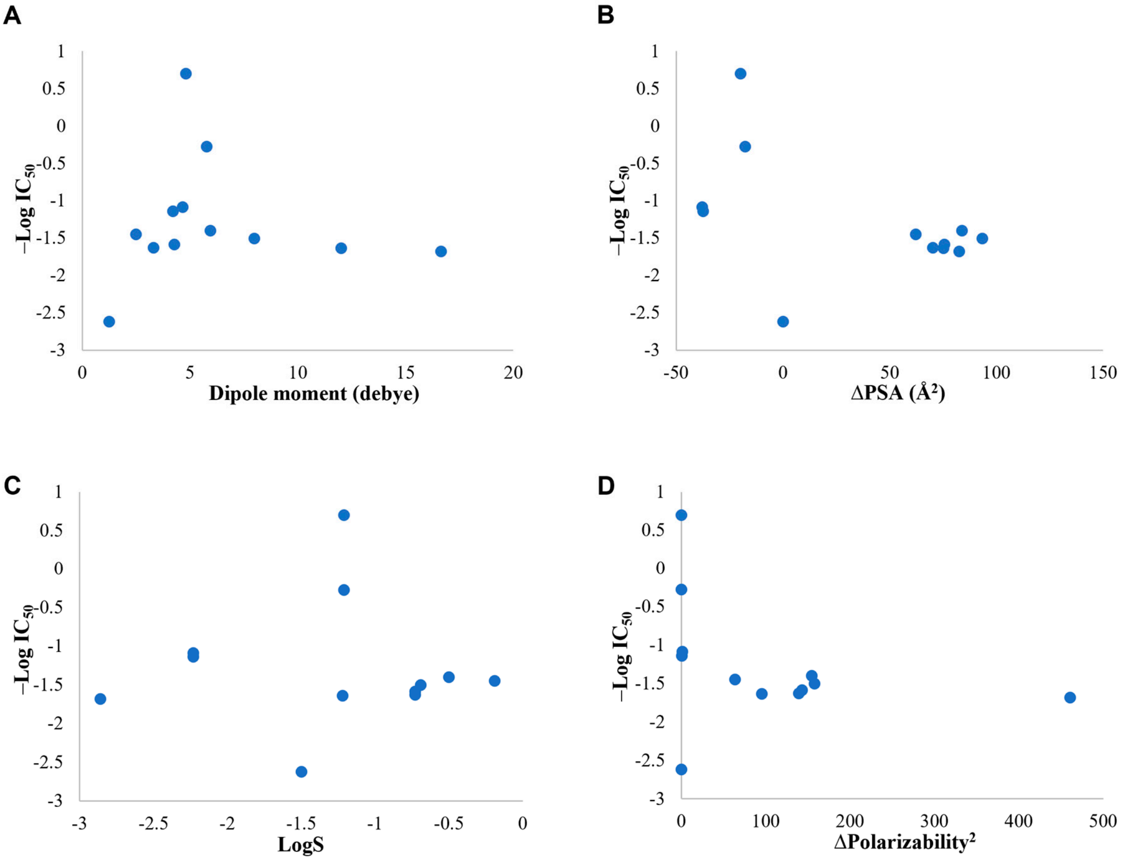

Figure S10. Independent correlations between the biological activity and the descriptors of the QSAR model. (A) –Log IC_{50} vs Dipole moment, (B) –Log IC_{50} vs ΔPSA, (C) –Log IC_{50} vs LogS, (D) –Log IC_{50} vs ΔPolarizability^2.

Table S1. Pearson correlation matrix.

Variables	**Dipole moment**	**ΔPSA**	**LogS**	**ΔPolarizability^2**
**Dipole moment**	1	0.1818	0.2138	0.5893
**ΔPSA**	0.1818	1	0.1994	0.4912
**Log S**	0.2138	0.1994	1	0.0628
**ΔPolarizability^2**	0.5893	0.4912	0.0628	1

Table S2. pKa values of the acid iridoids.

Ligand	*pKa*
Geniposidic acid	4.22
10-O-(E)-p-coumaroylgeniposidic acid	4.16
D1	4.51
D4	4.56
D7	4.56
D10	4.62
D29	4.72

Table S3. Molecular descriptors of designed iridoids. *Cont.*

**Ligand**	**$GAP_{HOMO-LUMO}$ (kcal)**	**Dipole moment (debye)**	**Area (Å²)**	**Volume (Å³)**	**PSA (Å²)**	**Ovality**	**LogP**	**Polarizability**	**HBD Count**	**HBA Count**
D1*	-125.85	1.81	217.56	199.31	78.714	1.32	-0.52	56.25	3	4
D2	-120.73	2.64	208.92	192.24	59.084	1.30	-0.79	55.73	2	4
D3	-127.72	3.02	244.68	223.58	65.992	1.37	0.14	58.20	2	4
D4*	-126.69	3.41	221.65	203.47	78.335	1.32	-0.12	56.58	3	4
D5	-123.30	5.40	214.52	196.18	61.021	1.31	-0.38	56.02	2	4
D6	-126.95	3.29	245.94	224.18	65.825	1.38	0.14	58.26	2	4
D7*	-126.50	3.73	223.82	203.34	80.250	1.34	-0.12	56.57	3	4
D8	-122.27	5.42	215.72	196.31	61.345	1.32	-0.38	56.04	2	4
D9	-123.61	4.80	187.58	170.55	42.052	1.26	0.42	53.94	1	3
D10*	-599.13	12.32	190.51	175.42	54.222	1.26	--	54.14	1	4
D11	-127.89	2.20	219.28	198.11	47.446	1.33	0.95	56.13	1	3
D12	-122.45	3.61	206.94	189.55	42.020	1.30	-0.08	55.49	1	3
D13	-123.80	3.20	210.58	193.53	41.979	1.30	0.32	55.80	1	3
D14	-122.68	4.37	211.90	193.67	41.090	1.31	0.32	55.83	1	3
D15	-122.56	3.47	218.12	199.03	41.939	1.32	0.44	56.26	1	3
D16	-123.82	2.79	221.00	203.00	41.821	1.32	0.84	56.57	1	3
D17	-122.67	4.42	222.08	202.97	40.996	1.33	0.84	56.58	1	3
D18	-122.73	4.10	216.01	196.71	62.142	1.32	-0.41	56.07	2	4
D19	-123.30	6.44	219.60	200.68	60.798	1.32	-0.01	56.39	2	4
D20	-123.12	5.94	220.74	200.82	61.263	1.33	-0.01	56.40	2	4
D21	-127.06	2.47	222.59	202.35	84.953	1.34	-1.17	56.48	3	5
D22	-127.83	3.60	226.70	206.31	83.982	1.34	-0.77	56.80	3	5
D23	-129.51	5.38	227.87	206.45	84.829	1.35	-0.77	56.79	3	5
D24	-120.42	2.63	229.19	210.22	61.867	1.34	-0.01	57.19	2	4

*These iridoids were analyzed in their anion form. --: Not determined with Spartan'20.

Table S3. Molecular descriptors of designed iridoids. *Cont.*

Ligand	GAP$_{HOMO-LUMO}$ (kcal)	Dipole moment (debye)	Area (Å^2)	Volume (Å^3)	PSA (Å^2)	Ovality	LogP	Polarizability	HBD Count	HBA Count
D25	-120.85	4.28	232.76	214.18	60.492	1.34	0.39	57.51	2	4
D26	-120.23	4.83	233.82	214.32	60.926	1.35	0.39	57.33	2	4
D27	-139.77	0.59	215.39	194.54	47.457	1.33	0.88	55.72	2	3
D28	-123.43	4.80	206.65	188.8	42.041	1.30	0.75	55.42	1	3
D29*	-89.18	11.93	211.04	193.89	55.479	1.30	--	56.19	1	4
D30	-126.98	2.29	237.49	216.71	46.402	1.36	1.28	57.65	1	3
D31	-128.21	3.95	219.07	198.98	65.082	1.33	0.36	56.20	2	4
D32	-128.28	4.00	199.75	180.67	64.951	1.29	0.03	54.71	2	4
D33	-121.04	4.05	224.89	206.79	41.505	1.33	1.52	56.91	1	3
D34	-121.19	4.05	205.95	188.56	41.547	1.30	1.19	55.43	1	3
D35	-123.99	3.36	197.34	178.62	60.872	1.29	-0.67	54.59	2	4
D36	-138.78	0.69	196.25	176.32	47.478	1.29	0.55	54.25	2	3
D37	-118.35	2.24	213.18	194.21	41.271	1.31	1.20	55.91	1	3
D38	-505.46	2.89	258.12	237.65	40.393	1.39	2.09	59.41	1	3
D39	-121.33	2.90	227.28	207.57	40.391	1.34	1.84	56.97	1	3
D40	-122.19	3.23	244.69	225.37	40.061	1.37	2.17	58.40	1	3
D41	-120.24	3.53	233.02	212.41	40.307	1.35	1.49	57.37	1	3
D42	-120.89	3.85	250.54	230.24	39.951	1.38	1.82	58.81	1	3
D43	-119.35	4.24	238.48	217.09	40.325	1.37	1.62	57.76	1	3
D44	-120.14	4.76	256.53	235.02	40.276	1.39	1.95	59.21	1	3
D45	-118.8	5.60	243.29	220.98	41.009	1.38	2.28	58.08	1	3
D46	-118.78	6.57	261.08	239.07	40.651	1.40	2.61	59.55	1	3
D47	-121.41	2.78	247.40	225.98	40.377	1.38	2.26	58.46	1	3
D48	-122.26	3.13	264.90	243.79	40.089	1.40	2.59	59.90	1	3
D49	-120.70	3.00	236.69	218.98	41.081	1.35	1.91	57.90	1	3
D50	-121.47	3.38	254.60	236.87	40.669	1.38	2.24	59.34	1	3
D51	-121.22	2.81	250.72	234.64	40.355	1.36	2.33	59.16	1	3
D52	-122.30	3.16	268.57	252.58	40.051	1.39	2.66	60.61	1	3
D53	-122.14	2.92	265.73	250.8	39.881	1.38	2.75	60.47	1	3
D54	-123.62	3.15	284.17	268.82	40.524	1.41	3.08	61.91	1	3
D55	-139.52	1.70	196.35	176.40	47.351	1.29	0.61	54.25	2	3
D56	-118.90	4.81	213.74	196.37	57.202	1.31	-0.41	56.08	2	4
D57	-119.30	4.54	221.22	203.82	76.266	1.32	-1.54	56.68	3	5
D58	-140.01	1.99	219.39	201.10	61.709	1.32	-0.28	56.25	3	4
D59	-137.43	2.62	226.10	208.66	80.151	1.33	-1.41	56.89	4	5
D60	-144.58	2.30	201.54	184.95	73.770	1.28	-1.50	54.89	3	5
D61	-145.63	3.19	207.72	190.41	80.817	1.30	-1.25	55.33	4	5
D62	-135.25	0.86	196.11	178.97	61.665	1.28	-0.79	54.50	3	4

*These iridoids were analyzed in their anion form. --: Not determined with Spartan'20.

Table S3. Molecular descriptors of designed iridoids. *Cont.*

Ligand	$GAP_{HOMO-LUMO}$ (kcal)	Dipole moment (debye)	Area (Å²)	Volume (Å³)	PSA (Å²)	Ovality	LogP	Polarizability	HBD Count	HBA Count
D63	-505.46	2.89	258.12	237.65	40.393	1.39	2.09	59.41	1	3
D64	-121.91	3.11	276.76	255.71	40.739	1.42	2.42	60.87	1	3
D65	-121.14	2.80	273.51	253.54	40.435	1.41	2.51	60.70	1	3
D66	-122.09	2.95	292.14	271.56	40.779	1.44	2.84	62.15	1	3
D67	-121.09	2.70	288.39	269.65	40.352	1.43	2.93	62.01	1	3
D68	-122.08	3.01	305.93	287.51	40.105	1.45	3.26	63.44	1	3
D69	-130.53	3.51	269.44	248.10	50.269	1.41	0.98	60.16	2	4
D70	-130.04	3.66	288.14	266.22	49.777	1.44	1.31	61.64	2	4
D71	-137.74	2.13	225.54	202.41	52.922	1.35	0.27	56.38	2	4
D72	-133.99	4.17	241.33	219.48	51.970	1.37	0.60	57.80	2	4
D73	-140.90	4.11	230.88	207.20	52.801	1.36	0.48	56.74	2	4
D74	-137.00	4.34	249.84	225.37	52.539	1.40	0.81	58.25	2	4
D75	-129.17	4.96	233.03	210.51	51.527	1.36	0.98	57.13	2	4
D76	-135.26	4.02	255.21	229.88	52.563	1.41	1.31	58.63	2	4
D77	-136.40	5.49	239.97	215.68	51.589	1.38	--	57.47	2	4
D78	-533.02	6.25	257.40	233.22	51.275	1.40	--	58.99	2	4
D79	-137.70	2.26	246.55	221.08	52.731	1.39	0.61	57.90	2	4
D80	-140.36	3.98	246.76	239.11	52.530	1.42	0.94	59.33	2	4
D81	-129.02	3.56	253.51	232.11	50.210	1.39	0.56	58.88	2	4
D82	-540.05	3.67	272.36	250.18	50.028	1.42	0.89	60.35	2	4
D83	-130.53	3.51	269.44	248.10	50.269	1.41	0.98	60.16	2	4
D84	-130.04	3.66	288.14	266.22	49.777	1.44	1.31	61.64	2	4
D85	-138.22	4.33	286.35	264.62	51.888	1.44	1.40	61.42	2	4
D86	-138.33	4.29	305.36	282.82	51.735	1.47	1.73	62.90	2	4

*These iridoids were analyzed in their anion form. --: Not determined with Spartan'20.
